# Crystal Structure, Theoretical Analysis, and Protein/DNA Binding Activity of Iron(III) Complex Containing Differently Protonated Pyridoxal–*S*-Methyl-Isothiosemicarbazone Ligands

**DOI:** 10.3390/ijms25137058

**Published:** 2024-06-27

**Authors:** Violeta Jevtovic, Luka Golubović, Badriah Alshammari, Maha Raghyan Alshammari, Sahar Y. Rajeh, Maha Awjan Alreshidi, Odeh A. O. Alshammari, Aleksandra Rakić, Dušan Dimić

**Affiliations:** 1 Department of Chemistry, College of Science, University Ha’il, Ha’il 81451, Saudi Arabia; 2 Faculty of Physical Chemistry, University of Belgrade, Studentski trg 12-16, 11000 Belgrade, Serbia

**Keywords:** DFT, BSA, HSA, pyridoxal–isothiosemicarbazone, QTAIM, crystal structure

## Abstract

Pyridoxal–*S*-methyl-isothiosemicarbazone (PLITSC) is a member of an important group of ligands characterized by different complexation modes to various transition metals. In this contribution, a new complex containing two differently protonated PLITSC ligands ([Fe(PLITSC–H)(PLITSC)]SO_4_)∙2.5H_2_O was obtained. The crystal structure was solved by the X-ray analysis and used further for the optimization at B3LYP/6-311++G(d,p)(H,C,N,O,S)/def2-TZVP(Fe) level of theory. Changes in the interaction strength and bond distance due to protonation were observed upon examination by the Quantum Theory of Atoms in Molecules. The protein binding affinity of [Fe(PLITSC–H)(PLITSC)]SO_4_ towards transport proteins (Bovine Serum Albumin (BSA) and Human Serum Albumin (HSA)) was investigated by the spectrofluorimetric titration and molecular docking. The interactions with the active pocket containing fluorescent amino acids were examined in detail, which explained the fluorescence quenching. The interactions between complex and DNA were followed by the ethidium-bromide displacement titration and molecular docking. The binding along the minor groove was the dominant process involving complex in the proximity of DNA.

## 1. Introduction

Pyridoxal–thiocarbazones are a class of ligands containing N, O, and S donor atoms. These compounds, characterized by different functional groups, are obtained by condensing ketone or aldehyde with thiosemicarbazide; therefore, they can be considered Schiff bases [1]. Due to the presence of various donor atoms, the binding mode can significantly differ depending on the protonation/deprotonation and stabilization of isomers [2,3,4,5].

From the pioneering works of Italian chemists gathered around Prof. Pelizzi’s group, in the 1980s [6,7], it was evident that ligand systems based on pyridoxal–carbazone would further develop. Initially, these ligand systems seemed promising in their ability to coordinate with transition metals and form complexes with pronounced biological and catalytic activity.

**Scheme 1:**
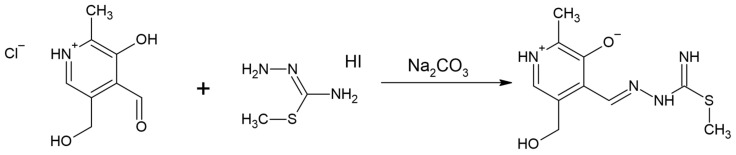
Synthesis of pyridoxal–isothiosemicarbazone (PLITSC).

**Scheme 2:**
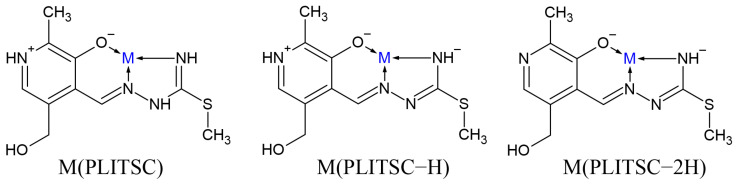
Binding modes of differently protonated PLITSC (M is metal).

The ligand pyridoxal–*S*-methylisothiosemicarbazone (PLITSC) is formed in the reaction between pyridoxal and *S*-methyl-isothiosemicarbazone, according to Scheme 1. The ability of sulfur to donate a lone electron pair to a central metal ion differentiates the thiosemicarbazone and isothiosemicarbazone ligands [8]. Condensation leads to the formation of Schiff base ligands. Ligand PLITSC is a tridentate ligand with coordination sites, which include the oxygen of phenolic hydroxyl, hydrazine nitrogen, and amide nitrogen, creating the ONN form of the coordinated ligand. Three forms of PLITSC can be found in complexes: neutral, mono-, and dianionic (Scheme 2). The monoanionic form is formed by the deprotonation of the hydrazine nitrogen atom, while in the dianionic form, the deprotonation of the pyridine nitrogen atom occurs.

Many transition metal complexes with PLITSC ligands are described in the literature [9,10,11,12,13,14]. These compounds have shown antimicrobial, anti-inflammatory, antioxidant, and antitumor activity [15,16]. In the last decade, several papers examined the catalytic properties of these compounds [17,18,19,20,21]. When iron complexes with PLITSC ligand are concerned, the preparation of several mono- and bis(ligand) and dicationic compounds are reported [22,23,24]. Determining the oxidation state of metals by XPS is important for discussing structural features and the compound’s reactivity, as shown in [25].

The binding affinity of compounds towards transport proteins (Human Serum Albumin (HSA) and Bovine Serum Albumin (BSA)) and DNA is often examined as the preliminary step in the assessment of the biological activity of compounds [26,27]. Transport proteins are essential for distributing fatty acids, metal ions, drugs, and toxins [28,29]. Fluorescence in BSA and HSA molecules predominantly arises from three amino acids: tryptophan, tyrosine (Tyr), and phenylalanine (Phe) [30]. Notably, tryptophan contributes to approximately 91% of the fluorescence signal. Excitation at a wavelength of 280 nm stimulates both tryptophan and tyrosine residues, while a wavelength of 295 nm selectively excites tryptophan residues alone [31]. Consequently, using the 295 nm excitation wavelength in spectrofluorimetric measurements proves convenient for targeting tryptophan residues specifically. On the other side, interaction with DNA is one of the most important pathways for the cytotoxic activity of compounds [32]. Transition metal complexes with ligands containing heterocyclic compounds show high binding affinity towards these proteins and DNA, resulting in considerable cytotoxicity towards certain cancer types [1,33].

The paper aims to present the synthesis and crystal structure of [Fe(PLITSC–H)(PLITSC)]^2+^ complex, which is particularly interesting due to the presence of two differently protonated ligand molecules. The Hirshfeld surface analysis was applied to examine the intermolecular interactions responsible for the structure’s overall stability. The theoretical structure analysis was performed on the optimized structure at B3LYP/6-311++G(d,p)(H,C,N,O,S)/def2-TZVP(Fe) level of theory. The Quantum Theory of Atoms in Molecules (QTAIM) approach was used to identify and quantify the intramolecular interactions between donor atoms and central metal ions and examine the changes in these interactions upon protonation. The protein and DNA binding affinities of the compound were investigated through spectrofluorimetric titration, while interactions at the molecular level were explained by molecular docking simulations.

## 2. Results and Discussion

### 2.1. Preparation of [Fe(PLITSC–H)(PLITSC)]SO_4_

PLITSC ligand was prepared according to the procedure presented in [32]. The complex compound was obtained after mixing ligand and FeSO4 dissolved in water. The crystallization occurred by slow evaporation, forming dark red–orange crystals. The structure of the obtained crystals was examined by the X-ray diffraction experiment, as explained below. The obtained compound was soluble in chloroform, dimethylsulfoxide, acetone, and acetonitrile, moderately soluble in methanol, ethanol, and water, and insoluble in diethyl ether and toluene.

### 2.2. Crystalographic Structure Analysis

**Figure 1:**
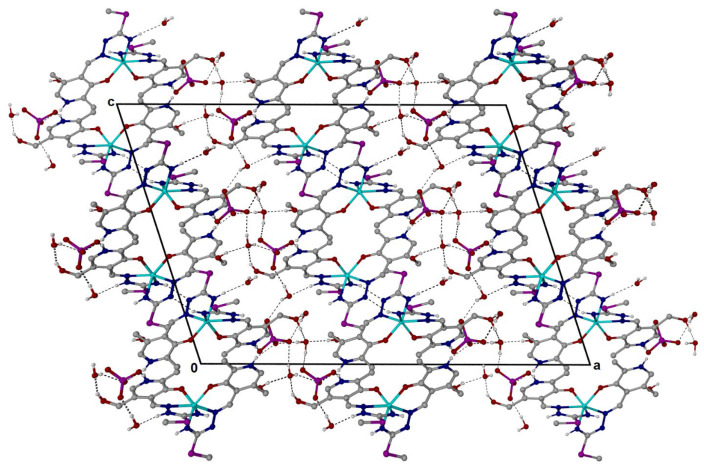
Cell packing as viewed along the b-axis, and showing the complex 3-D network of [Fe(PLITSC–H)(PLITSC)]^2+^ and SO_4_^2−^ ions formed through a variety of N-H∙∙∙X (X = O/N) and O-H∙∙∙O hydrogen bonds.

The structure of the newly synthesized Fe(III) complex with pyridoxal–isothiosemicarbazone proved very interesting from the structural analysis point of view. The crystal unit cell is characterized by two independent complex ions, surrounded by two counterions and five water molecules, which leads to the formula [Fe(PLITSC–H)(PLITSC)]_2_(SO_4_)_2_∙5H_2_O. In the packing, however, there are four unit cells, so a total of eight complex molecules are present (Figure 1). The atomic coordinates, bond lengths and angles, and anisotropic displacement parameters are presented in Tables S1–S3.

**Figure 2:**
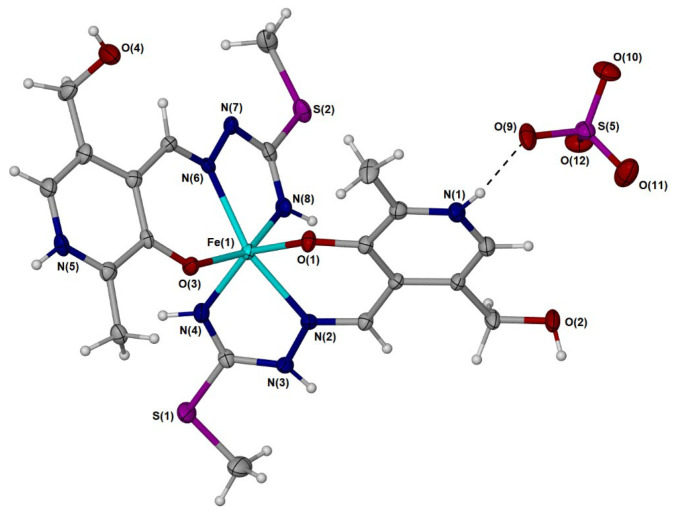
Molecular diagram of one of two unique [Fe(PLITSC–H)(PLITSC)]SO_4_ with non-hydrogen atoms represented by 50% displacement ellipsoids and hydrogen atoms as spheres of arbitrary size. Water molecules have been omitted for clarity. The metal center is Fe(III) with one neutral PLITSC ligand and one anionic PLITSC–H ligand, consistent with the red–orange color of the material.

The [Fe(PLITSC–H)(PLITSC)]^2+^ complex is a bis-ligand iron(III) complex with two PLITSC coordinated ligands, one in neutral and the other in anionic form (Figure 2), and sulfate group (SO_4_^2−^), which leads to the neutral charge of the compound. Two pyridine nitrogen atoms are protonated, while only one hydrazine nitrogen contains a directly bound hydrogen atom (Figure 2), which corresponds to two binding modes, according to Scheme 2. The synthesis was performed in water, resulting in five water molecules within the crystal structure.

The environment around the central Fe(III) is pseudo-octahedral due to the presence of two ONN PLITSC ligands. However, this geometry is slightly distorted due to interactions with solvent molecules and counterions. The rigidity of the Schiff base also influences the overall geometry and deviance from the perfect octahedral angles. The lengths (Table S2) are similar for the corresponding Fe-O and Fe-N interactions from the two molecules that form the independent part. A large number of hydrogen bonds are also present in structures, as shown in Table S5 and Figure 1. Hydrogen bonds are formed between water molecules and electronegative groups, such as hydroxyl, protonated hydrazine, and pyridine (Tables S4 and S5). The hydrogen bond-motif descriptors and symmetry transformations are presented in Table S5. The hydrogen bond lengths are between 2.659 and 2.931 Å, as the distance between donor and acceptor. These interactions are important for the overall stability of crystal structure.

### 2.3. Theoretical Structural and QTAIM Analysis of [Fe(PLITSC–H)(PLITSC)]^2+^

**Figure 3:**
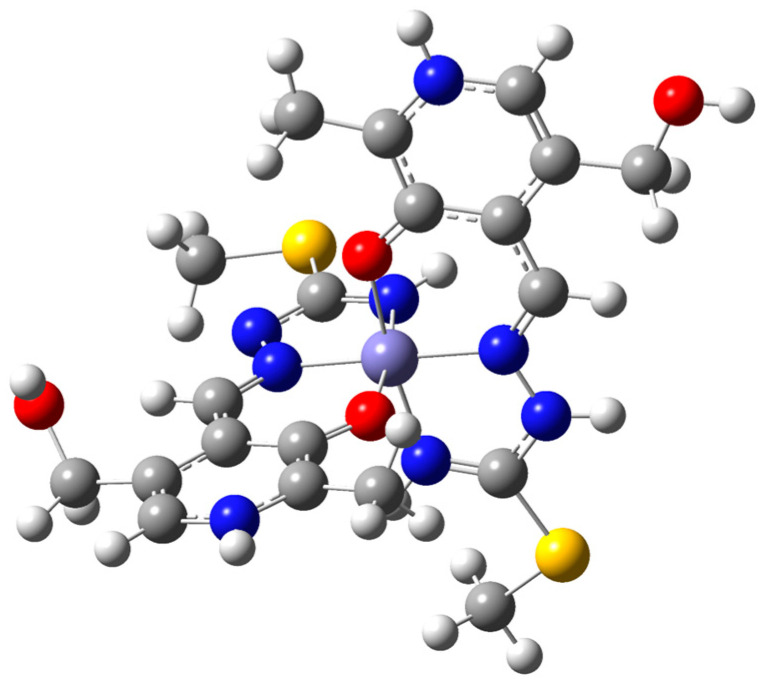
Optimized structure (at B3LYP/6-311++G(d,p)(H,C,N,O,S)/def2-TZVP(Fe) level of theory) of [Fe(PLITSC–H)(PLITSC)]^2+^ (Hydrogen-white, carbon-gray, nitrogen-blue, oxygen-red, iron-pale lilac).

The structure of complex cation was optimized at B3LYP/6-311++G(d,p)(H,C,N,O,S)/def2-TZVP(Fe) level of theory starting from the crystal structure without any geometrical constraints (Figure 3 and Tables S6 and S7). The bond lengths and angles in theoretical and experimental structures were compared by calculating the correlation coefficient and the mean absolute error (MAE), as previously shown in references [34,35]. The correlation coefficients are 0.98 (bond lengths) and 0.91 (bond angles). The MAE values for bond lengths and angles are 0.03 Å and 4.4°, proving that the selected theory level was appropriate for describing the obtained complex cation.

The bond lengths between iron and oxygen atoms are 1.95 and 1.91 Å in the experimental and 1.95 and 1.96 Å in the optimized structure. On the other hand, the lengths between iron and amino nitrogen atoms are 2.06 and 2.015 Å in protonated and deprotonated PLITSC ligands, while between iron and hydrazine nitrogen, 2.21 and 2.17 Å. These results show that larger distances were found between donor atoms of protonated PLITSC and iron, and it can be expected that these differences are reflected in the other structural parameters. Upon optimization, the bond lengths between iron and nitrogen atoms are between 1.88 and 1.97 Å. The amino nitrogen atoms are still closer to the central metal ion, although differences are less pronounced than in the experimental structure. This can be explained by the overall stabilization of the system and the absence of co-crystalized solvent molecules, counterions, and other complex cations in the unit cell. When protonation of hydrazine moiety occurs, the bond distance between neighboring nitrogen atoms only slightly increases by 0.02 Å in the experimental structure. The differences in distances between the aromatic ring’s deprotonated oxygen and carbon atoms are negligible and not influenced by deprotonation. Due to the rigidity of the structure and extended delocalization, other bond lengths are also not influenced by the addition of a proton.

The differences in bond angles are more pronounced after the optimization, and overall, the structure adopts angles characteristic of the octahedral geometry, leading to a lower correlation coefficient. For example, the bond angles between oxygen, iron, and amino/hydrazine nitrogen atoms in the experimental structure are 144.2/79.9, while in the optimized, 173.0/91.9°, which explains that pseudo-octahedral geometry is a consequence of the interaction within crystal packaging, similar to previous finding on complexes with this groups of ligands [36,37]. The angles formed between two oxygen atoms and central metal ions are 89.4 and 90.6° in crystal and the theoretical structure, respectively. Again, much lower differences upon optimization were found for the deprotonated structure. This is a consequence of the formed interactions between protonated hydrazine nitrogen with water molecules and sulfate ions, as explained in the previous section. These interactions are also present when C–N–N of the bridgeing groups are examined. A larger angle of 116.6 compared to 110.0° was obtained for the protonated structure.

The investigation of the stability interactions within complex cations is limited to the interactions between donor atoms and central metal ions and other bonds influenced directly by the protonation. The stabilization interactions are enlisted in Table 1, together with the electron density and Laplacian from the QTAIM analysis. The interactions between donor atoms and iron ions can be classified as open shell interactions, although electron densities have values that are around 0.1 a.u. These interactions between the oxygen atom and iron ion have an electron density of 0.090 a.u. and Laplacian of around 0.500 a.u. Electron density is higher when hydrazine and amino nitrogen atoms are concerned, especially in the case of amino nitrogen atoms. This is expected, as there is an extended delocalization within bridging atoms of PLITSC that include the lone electron pair of hydrazine nitrogen atoms, thus lowering its ability for electron donation to the central metal ion. It is important to observe that electron densities are lower when PLITSC structure is deprotonated, for example, 0.091 and 0.104 a.u. for the interactions of deprotonated and protonated PLITSC ligand hydrazine nitrogen atoms. This is a consequence of the overall ligand structure relaxation in the presence of hydrogen atoms. The distances between donor atoms and iron ions nicely reflect changes in electron density. Protonation of structure also influences the distances between atoms neighboring protonated nitrogen. The electron densities are lower in N–N_hydrazine_ and C=N_hydrazine_ bonds of the protonated structure (Table 1), while bond lengths are higher (1.38 vs. 1.33 Å for C=N_hydrazine_). These results explain that the protonation of structure is important for the binding properties of investigated ligands and further determines the stability of complex compounds.

### 2.4. BSA Protein Binding Affinity of [Fe(PLITSC–H)(PLITSC)]SO_4_

**Figure 4:**
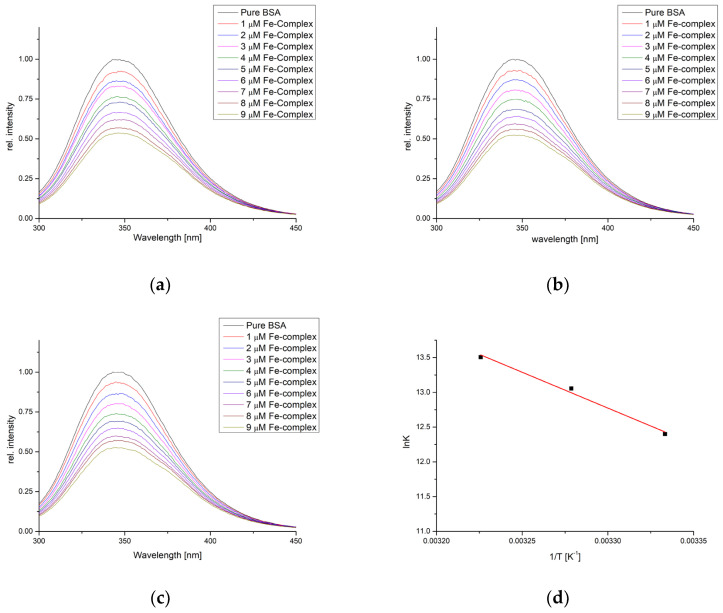
Fluorescence emission spectra of BSA for the titration with [Fe(PLITSC–H)(PLITSC)]SO_4_ at (**a**) 27°, (**b**) 32°, (**c**) 37°, and (**d**) Van ’t Hoff plot for the binding process.

The protein binding affinity of the complex towards BSA was analyzed by spectrofluorimetric titration. This protein contains fluorescent amino acids in the active pockets, and their emission is initiated by the excitation wavelength of 280 nm. Upon a change in the chemical environment of the amino acids, the fluorescence is quenched by the presence of a complex compound. Figure 4 presents the emission spectra of BSA with the increased concentrations of compound, and the thermodynamic parameters of binding are enlisted in Table 2.

Upon addition of the complex, the fluorescence intensity decreased in a concentration-dependent manner. This dependence followed a double-log Stern–Volmer quenching mechanism. The correlation coefficients are presented in Table 2, with the binding constants and the number of binding positions. The correlation coefficients are between 0.985 (27 °C) and 0.999 (37 °C) (Figure S1), with the number of binding positions around 1, proving that one complex molecule is bound to one BSA molecule. With increased temperature, the binding constants increase from 2.43 × 10^5^ to 7.35 × 10^5^ M^−1^.

The thermodynamic parameters obtained from previous results yielded a change in entropy and enthalpy of 85.72 kJ mol^−1^ and 389.06 J mol^−1^ K^−1^, respectively. The positive change in this process leads to the conclusion that the process is entropy-driven and that some of the rotational and translational degrees of freedom are lost upon binding. This is expected, as multiple groups of the ligand can form interactions with the surrounding amino acids. A similar was observed for the iron(III) complex containing pyridoxal–thiosemicarbazone ligand [37]. The changes in Gibbs free energy of binding are −31.0, −33.0, and −34.9 kJ mol^−1^ for 27, 32, and 37 °C. These values are slightly higher than those calculated for the previously mentioned complex with pyridoxal–thiosemicarbazone ligand, probably because ligands surrounding [Fe(PLITSC)(PLITSC-H)]^2+^ form a multitude of interactions with amino acids.

The interactions between complex and BSA were further examined by the molecular docking simulations in the following section.

### 2.5. HSA Protein Binding Affinity of [Fe(PLITSC–H)(PLITSC)]SO_4_

**Figure 5:**
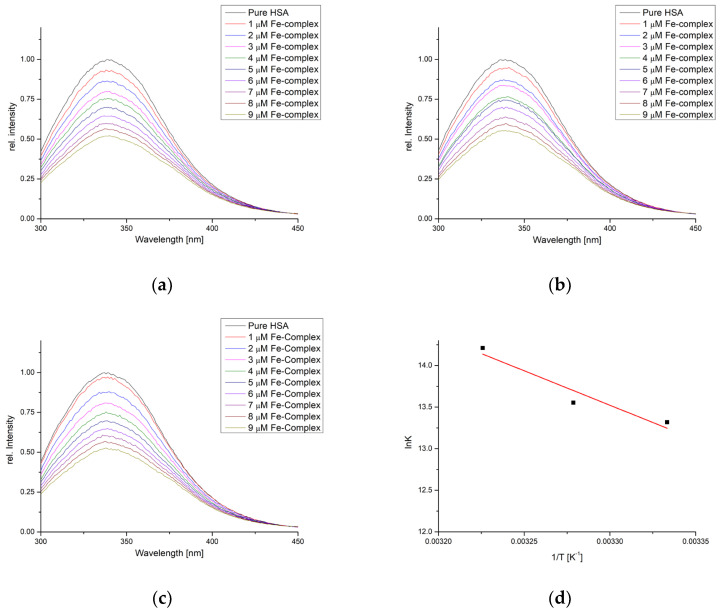
Fluorescence emission spectra of HSA for the titration with [Fe(PLITSC–H)(PLITSC)]SO_4_ at (**a**) 27°, (**b**) 32°, (**c**) 37°, and (**d**) Van ’t Hoff plot for the binding process.

The binding activity towards HSA was also examined using spectrofluorimetric titration in the same temperature range. The fluorescence intensity of HSA decreased when the complex was added following the double-log Stern–Volmer equation (Figure 5). The correlation coefficients for this dependency were between 0.982 and 0.999 (Figure S1 and Table 3). The number of binding positions was 1.16, 1.19, and 1.23, showing that one molecule of complex interacted with one HSA molecule. The binding constants were higher when compared to the same temperature in BSA experiments (6.08 × 10^5^ (27 °C), 7.69 × 10^5^ (32 °C), and 1.49 × 10^6^ M^−1^ (37 °C)). The binding constants increase with the increase in temperatures.

The changes in enthalpy and entropy of binding were 68.89 kJ mol^−1^ and 339.78 J mol^−1^ K^−1^, respectively, which led to the changes in Gibbs free energy of binding of −33.0, −34.7, and −36.4 kJ mol^−1^. These thermodynamic parameter values were comparable to those obtained when BSA binding affinity was examined. This is expected, as the active pockets of both proteins contain the same amino acids, as shown in the molecular docking study.

### 2.6. Molecular Docking Study towards BSA and HSA

The spectrofluorimetric measurements revealed a significant quenching of fluorescent emission from HSA and BSA when excited at 280 nm. Molecular docking simulations are thus undertaken to verify the binding of the [Fe(PLITSC–H)(PLITSC)]^2+^ complex in close proximity to tryptophan residues and elucidate the specific binding positions and intermolecular interactions at these sites. For each protein, ten positions were examined (Table S8 and Figure S2). The calculated binding affinities of the [Fe(PLITSC–H)(PLITSC)]^2+^ complex ion to HSA (−30.3 kJ mol^−1^) and BSA (−28.8 kJ mol^−1^) suggest the feasibility of its transportation by serum albumins within the circulatory system, underscoring its potential systemic distribution. However, to definitely confirm this claim, it is imperative to ascertain the precise binding sites of the [Fe(PLITSC–H)(PLITSC)]^2+^ complex within serum albumins.

**Figure 6:**
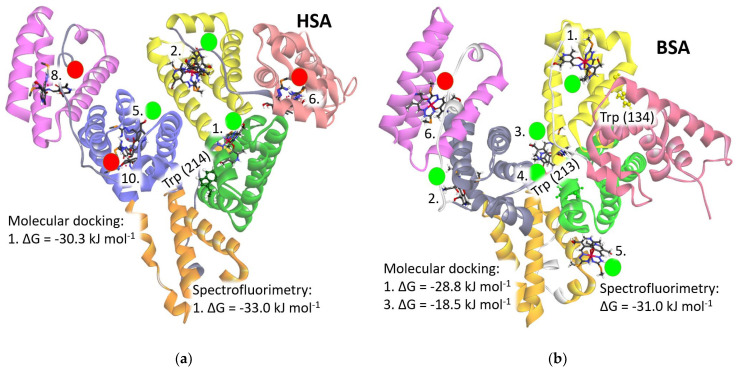
Structure of the [Fe(PLITSC–H)(PLITSC)]^2+^ complex (colored by element type) interacting with (**a**) Human Serum Albumin (HSA) and (**b**) Bovine Serum Albumin (BSA) molecules. Green circles with numbers indicate favorable binding positions with negative values of the change in Gibbs free energy of binding (ΔG), while positions marked with red circles denote unfavorable interactions with positive changes. The ranking of binding positions is denoted by assigned numbers on the structures, with lower numbers adjacent to binding sites exhibiting the highest binding energy. The subdomains of HSA and BSA are depicted in the following colors: IA in light reddish, IB in yellow, IIA in green, IIB in orange, IIIA in purple, and IIIB in pink.

The homology analysis of two serum albumins, HSA and BSA, indicates a high degree of similarity between them with minor discrepancies. The structure of albumins, as depicted in Figure 6, comprises three domains (I, II, and III), each subdivided into two subdomains, resulting in a total of six subdomains (IA, IB, IIA, IIB, IIIA, and IIIB), each depicted in distinct colors for clarity: IA (light reddish), IB (yellow), IIA (green), IIB (orange), IIIA (purple), and IIIB (pink). Peptide chains (depicted in light gray) connect two subdomains within each domain. The fatty acid binding sites (FA), crucial for fatty acid transportation, are predominantly situated within the cavities of the subdomains. Notably, Trp214 in HSA and Trp213 in BSA are positioned between subdomains IIA and IIB at the rear of the serum albumin molecule. This region accommodates one fatty acid binding site (FA8) and an additional site (FA9) positioned above it.

**Figure 7:**
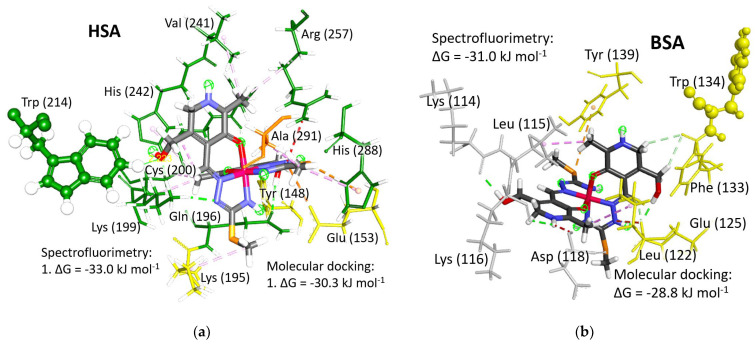
The optimal binding positions of [Fe(PLITSC–H)(PLITSC)]^2+^ (with the structure colored by element type) in (**a**) HSA (FA9 in IB subdomain, near Trp214) and (**b**) BSA (FA1 in IB subdomain, near Trp 134). Amino acid residues are color-coded according to the subdomain to which they belong: light gray for the peptide chain between subdomains, yellow for the IB subdomain, and green for the IIA subdomain.

Five out of the ten binding positions could be the binding sites for the [Fe(PLITSC–H)(PLITSC)]^2+^ complex, as evidenced by a negative change in Gibbs free energy of binding (ΔG_bind_) (Table S8). Conversely, the remaining five binding modes, highlighted with red circles in Figure 6, are deemed implausible due to their positive change in Gibbs free energy of binding values. Notably, the optimal binding energy is achieved proximal to the Trp214 residue within the FA9 binding site of the HSA molecule, where the change in Gibbs free energy of binding amounts to −30.3 kJ mol^−1^, closely resembling the experimentally obtained value of −33.0 kJ mol^−1^. Similarly, the prime binding position in BSA, located at binding site FA1 within the IB subdomain adjacent to Trp134 residue, exhibits a change in Gibbs free energy of binding of −28.8 kJ mol^−1^, in close agreement with the experimental value of −31.0 kJ mol^−1^. Figure 7 illustrates the optimal binding sites in both HSA and BSA, showcasing their respective interactions with surrounding amino acids in detail. The second-best binding energies in both proteins are notably lower, approximately 5 kJ mol^−1^ (Table S8). Therefore, it can be discussed that the [Fe(PLITSC–H)(PLITSC)]^2+^ complex will primarily bind to positions designated as “1” in Figure 6, aligning energetically with the findings from quenching experiments. Such circumstances, achievable in vitro under experimental conditions with elevated concentrations of the [Fe(PLITSC–H)(PLITSC)]^2+^ complex, are not reflective of physiological conditions within the circulating blood.

Serum albumins primarily function as carriers for fatty acids throughout the circulatory system, which is evident from their designation as fatty acid binding sites (FA). The ability of synthesized drugs to bind to FA binding sites and facilitate transport to target destinations represents a significant advancement in medical therapeutics and human welfare. In cases where the optimal binding site is occupied by a fatty acid molecule, the [Fe(PLITSC–H)(PLITSC)]^2+^ complex may bind to one of the subsequent four binding sites characterized by the negative Gibbs free energy of binding. Importantly, all FA binding sites are sufficiently shielded from the disruptive forces of blood flow within the circulation, ensuring secure retention of the [Fe(PLITSC–H)(PLITSC)]^2+^ complex for delivery to intended destinations. Detailed examination of the interactions between the Fe(III)-PLITSC complex and the surrounding environment at the optimal binding positions in HSA (Figure 7a) and BSA (Figure 7b) provides invaluable insights into the binding process.

HSA possesses only one tryptophan residue within its structure. The binding to the FA9 binding site within subdomain IB undoubtedly triggers the quenching of fluorescent emission. Conversely, BSA harbors two tryptophan molecules, with Trp134 situated within the IB subdomain, following the FA1 binding site, and another Trp213 located within the IIB subdomain, in close proximity to the FA8 binding site. However, considering that the binding affinity at the FA8 binding site (−18.5 kJ mol^−1^) is more than 10 kJ mol^−1^ lower than at the FA1 binding site (−28.8 kJ mol^−1^), the [Fe(PLITSC–H)(PLITSC)]^2+^ complex lacks the opportunity to bind to the FA8 binding site under experimental conditions (Table S8). Therefore, the binding of the [Fe(PLITSC–H)(PLITSC)]^2+^ complex to the FA1 binding site, in immediate proximity to Trp134, is the primary cause of the observed fluorescence quenching in BSA during the conducted spectrofluorimetric measurements.

In the case of HSA, the sole interaction with Trp214 involves a weak carbon-hydrogen bond formed between the oxygen atom in the OH group of the [Fe(PLITSC–H)(PLITSC)]^2+^ complex and a hydrogen atom attached to the aromatic ring of the Trp214 residue. The distance between the oxygen and hydrogen atoms in this carbon-hydrogen bond is a mere 2.223 Å. Similarly, the closest distance between the Trp134 residue and the [Fe(PLITSC–H)(PLITSC)]^2+^ complex in BSA amounts to 4.378 Å. This proximity is sufficiently close to impede fluorescent emission from the tryptophan residues.

The FA1 binding site is more spacious compared to the FA8 binding site. The [Fe(PLITSC–H)(PLITSC)]^2+^ complex has more contact with amino acid residues in HSA molecules than in BSA. Polar functional groups of the [Fe(PLITSC–H)(PLITSC)]^2+^ complex are less involved in electrostatic interactions, while hydrogen bonding and the hydrocarbon part of this compound are less available for hydrophobic interactions with the surroundings. Therefore, the energy release upon binding to BSA is lower.

The FA8 binding site of HSA exhibits a significantly broader array and greater quantity of interactions. Polar and charged groups within the [Fe(PLITSC–H)(PLITSC)]^2+^ complex engage in electrostatic interactions and hydrogen bonding. Hydrophobic interactions are facilitated by alkyl portions, with methyl groups substituted on aromatic rings playing a prominent role. Specifically, amino acids Lys199, Cys200, Ala241, Arg257, Cys245, and Ala241 engage in alkyl–alkyl interactions with these alkyl groups. Additionally, a π–alkyl interaction occurs with the His242 residue. The methyl group attached to sulfur engages in a singular hydrophobic interaction with Lys195. Hydroxyl groups readily form hydrogen bonds with Lys199, Trp214, Tyr148, and Gln195. The positively charged azo group engages in both electrostatic interactions and hydrogen bonding, notably with Glu153 and Lys199. The hydrogen attached to the aromatic carbon forms a hydrogen bond with Cys245, while the aromatic N-H group participates in a hydrogen bond with sulfur from Cys200.

### 2.7. DNA Binding Affinity of [Fe(PLITSC–H)(PLITSC)]SO_4_

The interactions between the obtained complex and DNA were investigated by the ethidium bromide (EB) displacement studies. EB (3,8-Diamino-5-ethyl-6-phenylphenanthridinium bromide) is a common indicator of intercalation [38]. In the reaction between EB fluorophore and nucleic acids, soluble complexes are formed with an excitation wavelength of 520 and an emission maximum of 600 nm. The intensification of the fluorescence is due to the intercalation of the planar phenenthridinium ring between adjacent base pairs on a double helix [39]. The changes in the fluorescence intensity are often used to study the interactions with other compounds with DNA through fluorescence quenching [40]. When in unbound form, EB does not show any measurable fluorescence due to the quenching by the solvent molecules [41].

**Figure 8:**
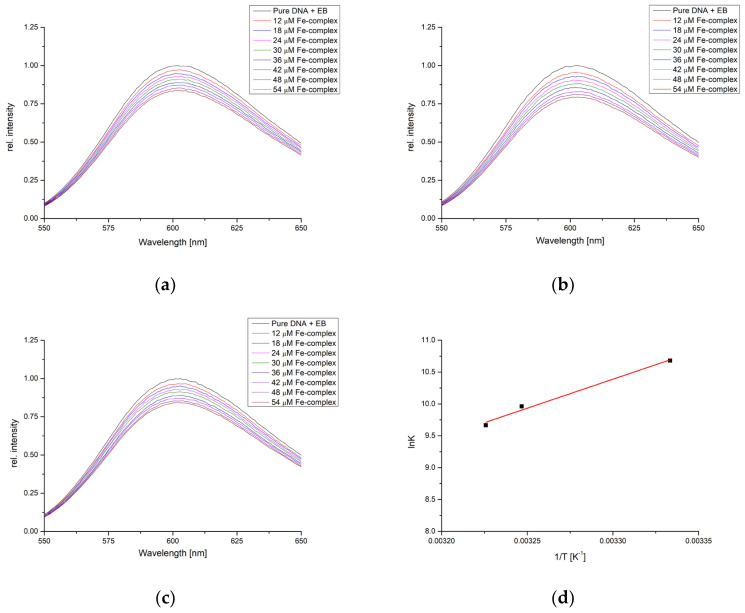
Fluorescence emission spectra of DNA-EB for the titration with [Fe(PLITSC–H)(PLITSC)]SO_4_ at (**a**) 27°, (**b**) 35°, (**c**) 37°, and (**d**) Van ’t Hoff plot for the binding process.

Figure 8 presents fluorescence spectra of EB-CT-DNA complexes in the phosphate buffer saline (pH = 7.4) for the solution containing 5 × 10^−5^ M of CT-DNA, determined by the molar absorption coefficient at 260 nm and 5 × 10^−6^ M of EB. The concentration of complex ranged from 1.2 to 5.4 × 10^−5^ M. The measurements were repeated at three temperatures (27, 35, and 37 °C) to obtain the thermodynamic parameters of binding. A decrease in fluorescence was observed for each complex addition, indicating the competition with EB in binding to DNA. These results prove that the obtained complex can react with the DNA molecules through intercalation. The double-log Stern–Volmer quenching equation was applied to the data (Figure S1), and the correlation coefficients, number of binding positions, and binding constants are presented in Table 4.

The number of binding positions for investigated complex and DNA is between 1.12 and 1.16, with binding constants of 4.37 × 10^4^ (27°), 2.00 × 10^4^ (35°), and 1.55 × 10^4^ M^−1^ (37°), which shows that with an increase in temperature, the binding constants decrease. This leads to the changes in enthalpy and entropy of binding of −78.7 kJ mol^−1^ and −173.4 J mol^−1^ K^−1^. The trend in these values is somewhat different from transport proteins, proving that stronger stabilization occurs through interactions between complex and nucleobases. The change in Gibbs free energy is negative, between −26.7 and −24.9 kJ mol^−1^, which signifies the spontaneity of binding. This could have important implications for the obtained compound’s possible biological activity and cytotoxicity.

The robust binding affinity, as found in the molecular docking simulations, of the [Fe(PLITSC–H)(PLITSC)]^2+^ complex to DNA, indicated by a binding energy of −26.7 kJ mol^−1^, suggests its potential to deactivate DNA molecules, thus presenting a promising avenue for its application as an anticancer agent. Further computational analysis aims to elucidate the specific DNA conformation to which the [Fe(PLITSC–H)(PLITSC)]^2+^ complex binds and the precise binding sites within the DNA molecule.

**Figure 9:**
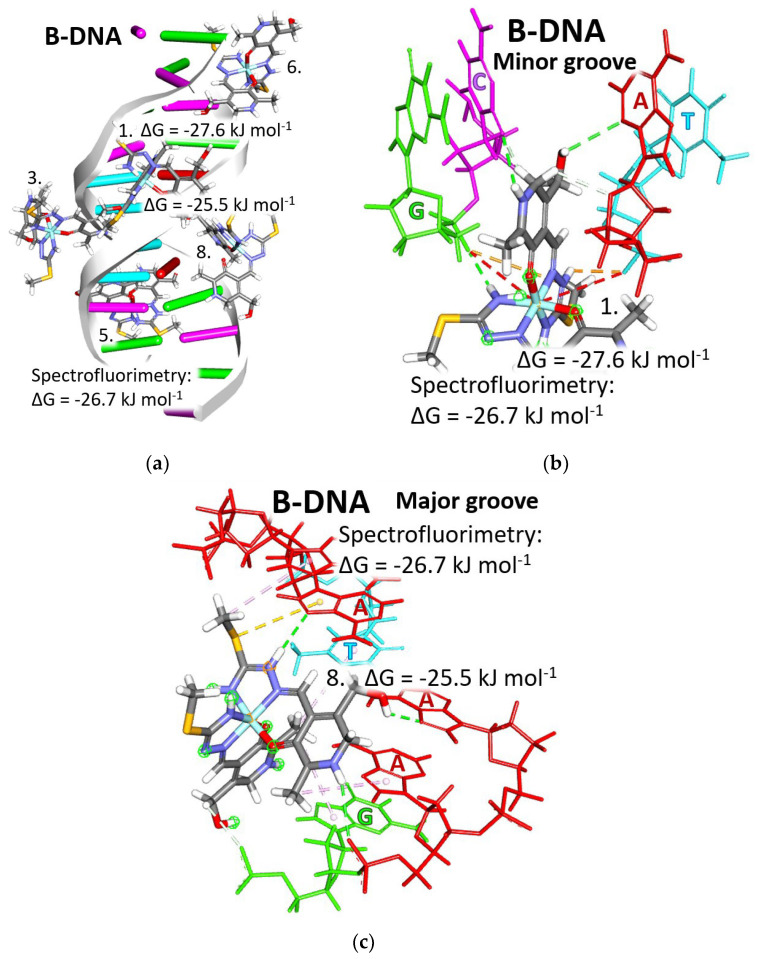
Positions of bound [Fe(PLITSC–H)(PLITSC)]SO_4_ complex (with the structure colored by element type) interacting with B-DNA (1-BNA) type: (**a**) all binding sites; (**b**) the most favorable binding site, 1, from the minor groove side; and (**c**) the least favorable binding site, 8, from the major groove side. Each nucleotide acid is represented by a different color: red for Adenine (A), green for Guanine (G), cyan for Cytosine (C), and yellow for Thymine (T). The two light gray strands represent the sugar–phosphate backbone.

The spectrofluorimetric measurements gave the value of the change Gibs free energy of binding for the complex to DNA structure to be −27.6 kJ mol^−1^, similar to the results obtained by molecular docking calculations. Calculations were performed on the B form of DNA (B-DNA) from the receptor, which is a double-strand containing four adenine-timin (A-T) base pairs in the middle of the structure, while four guanine-cytosine (G-C) base pairs are present on each side of the double strand (Figure 9a). B-DNA emerges as a prevailing conformation in biological systems, existing under conditions of physiological relevance, marked by elevated hydration levels and diminished salinity. This canonical DNA configuration showcases a right-handed double helical arrangement, where base pairs adopt an anti-conformation, thereby delineating minor and major grooves within the helical architecture.

The values of the change in Gibbs free energy of binding obtained by the molecular docking calculations do not defer much from the binding site to the binding site; the entire range of values is in the narrow interval between −27.6 and −25.1 kJ mol^−1^ (Table S8). The minimal difference in binding energies suggests that the [Fe(PLITSC–H)(PLITSC)]^2+^ complex binds to the B-DNA form practically at any binding site. Carefully analyzing the molecular docking results, it can be noticed that some factors favor slightly the binding at some binding sites over others. Among favorable factors are the type and number of nucleic acids involved, including sugars and phosphate groups in the interactions, and whether the complex is bound from the minor or major groove side. Good balance is achieved when the [Fe(PLITSC–H)(PLITSC)]^2+^ complex interacts with all types of nucleic acids equally and when sugar and phosphate groups are included.

The approach of the complex from the side of the minor groove opens the possibility that the phosphate backbone could be involved in the interactions. Electrostatic attractions of positively charged groups of the [Fe(PLITSC–H)(PLITSC)]^2+^ complex with the negatively charged phosphate groups and hydrogen bonds with sugar oxygen are the additional stabilizing effect for the adduct formed between the investigated complex and B-DNA. As is represented roughly in the whole B-DNA structure in Figure 9a and detail in Figure 9b, the binding of the [Fe(PLITSC–H)(PLITSC)]^2+^ complex at the binding site 1 meets all criteria. The [Fe(PLITSC–H)(PLITSC)]^2+^ complex approached the B-DNA from the minor groove in the double strand of α-helix that adopted the B conformation. Nucleic acids, sugar, and phosphate groups are closely packed from the minor grove side and easily accessible for interactions with a ligand. This enabled the Fe^3+^ cation, positively charged azo, and amide groups to establish strong electrostatic attraction with negatively charged phosphate groups. The sugar part of the DNA backbone forms hydrogen bonds with numerous polar groups of the [Fe(PLITSC–H)(PLITSC)]^2+^ complex. In binding site 1, the [Fe(PLITSC–H)(PLITSC)]^2+^ complex is inserted between A-T and G-C base pairs at an equal distance, having enough space to establish a good quality of hydrophobic, electrostatic interactions and hydrogen bonds with four nucleic acids. The complex bound to the binding site 1 has the lowest Gibbs free energy that can be achieved between B-DNA and the Fe(III)-PLITSC complex, 27.6 kJ mol^−1^.

The [Fe(PLITSC–H)(PLITSC)]^2+^ complex experiences few disadvantages when approaching from the major groove side. The two backbone strings are widely opened, which makes it impossible for [Fe(PLITSC–H)(PLITSC)]^2+^ complex to simultaneously form interactions with base pairs and the sugar-phosphate backbone. In particular, at binding site 8, the investigated complex interacts with base pairs unevenly with four adenines, guanine, and thymine. There is no electrostatic interaction with sugars or phosphate groups. The space is overcrowded with nucleic acids, so the obtained complex is forced to form mostly long-distance interactions with π systems of nucleic acid systems: π–S and π–alkyl interactions. The adenine prevails in interactions, disturbing the balance between nucleic acids. In this site, eight is bound one cluster since here establishes the lowest change in Gibbs free binding energy, −25.5 kJ mol^−1^.

## 3. Materials and Methods

### 3.1. Chemicals

All chemicals were obtained from commercial manufacturers and used without further purification. The ligand was prepared according to the previously described procedure [42].

### 3.2. Synthesis of [Fe(PLITSC–H)(PLITSC)]SO_4_

The amount of 0.01 mol of PLITSC ligand (2.54 g) was dissolved in 15 cm^3^ of water with heating, followed by 0.01 mol FeSO_4_ (1.52 g) dissolution in 15 cm^3^ H_2_O and addition to ligand solution. A clear dark solution was obtained and left at room temperature to crystallize by slow evaporation. After a few hours, dark red–orange crystals appeared. Yield: 0.20 g (75%).

### 3.3. X-ray Analysis

A representative red–orange thin plate crystal with dimensions 0.229 × 0.064 × 0.023 mm was selected and mounted on a nylon cryoloop. Diffraction data were collected at 123 K using CuKα radiation (λ = 1.54184 Å) on a Rigaku Synergy S diffractometer (Rigaku, Tokio, Japan) fitted with a HYPIX 6000 hybrid photon counting detector. Data were collected and processed, including an empirical (multi-scan) absorption correction, with the CrysAlisPro software [43]. The structure was solved and refined by standard methods using the SHELX software suite in conjunction with the Olex2 graphical interface [44,45]. Non-hydrogen atoms were refined with anisotropic displacement ellipsoids, and hydrogen atoms attached to carbon were placed in calculated positions using a riding model. The positions of hydrogen atoms attached to oxygen and nitrogen were apparent in the different Fourier maps. They were refined with restrained distances, d(O-H) = 0.88(2)Å or d(N-H) = 0.91(2)Å, and geometries (DFIX/DANG). The structure, as modeled in the non-centrosymmetric space group *Cc*, was refined to be a racemic twin (TWIN/BASF). The structure could also be solved and refined in the centrosymmetric space group *C*2/*c*, with one [Fe(LH_2_)(LH)]^2+^ cation, a disordered [SO_4_]^2−^ anion and 2.5 water molecules; however, the final R1 value of 0.1197 was significantly greater, and the current non-centrosymmetric model is preferred. The crystal structure was deposited in the Cambridge Crystallographic Data Centre (CCDC, 12 Union Road, Cambridge, UK; e-mail: depos-it@ccdc.cam.ac.uk); the CCDC deposition number of the compound is **2345683**. Crystallographic data and structure refinement are presented below in Table 5.

### 3.4. Theoretical Analysis

The structure of the investigated complex compound was optimized in the Gaussian 09 Program Package [46] starting from the experimental structure. The selected functional was B3LYP [47] in conjunction with 6-31++G(d,p) [10] basis set for non-metallic atoms (H,C,N,O,S) and def2-TZVP for iron. The selected theory level was previously used to describe similar compounds [48]. The optimization was performed without any geometrical constraints, and the minima on the potential energy surface were verified by the absence of imaginary frequencies. Several spin states were optimized, and based on the comparison with crystal structure parameters, the one resembling it the most was selected. The intramolecular interactions, responsible for the structure’s overall stability, were further examined by the Quantum Theory of Atoms in Molecules (QTAIM), which was also applied. This approach is based on Bader’s theory of Atoms in Molecules [49,50], and determination of the interaction type requires examination of the electron density and Laplacian within the Bond Critical Points (BCP) and Ring Critical Points (RCP) [51]. The closed-shell interactions (covalent bonds) are characterized by the electron density of 0.1 a.u. and large negative Laplacian, while open-shell interactions (ionic bonds, hydrogen bonds, and van der Waals interaction) have electron density between 0.001 and 0.04 a.u. and small positive Laplacian [52]. These calculations were performed in the AIMAll program package [53].

### 3.5. Spectrofluorimetric Measurements

The binding process to BSA and HSA was examined by the spectrofluorimetric titration on the Cary Eclipse MY2048CH03 instrument. The scan rate was set to 600 nm min^−1^ with both slits of 5 nm. The excitation wavelength was 295 and 280 nm for BSA and HSA, respectively. The emission wavelength range was between 310 and 500 nm. These excitation wavelengths correspond to the tryptophan residues found in the protein structure. The concentration of proteins was held constant at 5 × 10^−6^ M in 1 M phosphate saline (pH = 7.4). The concentration of the complex was between 1 and 10 × 10^−6^ M. The emission spectra were recorded two minutes after the addition of complexes. The same methodology was previously applied to different complexes with similar ligands [36,37]. The relative decrease of protein fluorescence intensity followed the double-log Stern–Volmer quenching equation:

(1) $$\log\left(\frac{I_{0}-I}{I}\right)=\log K_{b}+n\log\left[Q\right]$$

In the presented equation, *I*_0_ and *I* are the fluorescence emission intensities of BSA/HAS without and with added metal complexes, *K_b_* is the binding constant, *n* is the number of binding places within the protein structure, and [*Q*] is the concentration of metal complex responsible for the fluorescence quenching.

These measurements were repeated at three temperatures (27, 32, and 37 °C) to determine the change in enthalpy, entropy, and Gibbs free energy from the Van ’t Hoff plot:

(2) $$\ln K_{b}=-\frac{∆H_{b}}{RT}+\frac{∆S_{b}}{R}$$

The competitive DNA binding studies were performed by spectrofluorometric titration. The commercially available calf thymus DNA was utilized with 41.9 mol% G-C and 58.1 mol% A-T. The absorbance of CT-DNA at 260 nm is equivalent to 50 μg of double-stranded DNA. In these experiments, the ethidium bromide is replaced by the obtained complexes. CT-DNA and ethidium bromide concentrations were held constant at 5 × 10^−5^ and 5 × 10^−6^ M, respectively, in phosphate buffer saline (pH = 7.4). The interactions between complex and DNA included the successive addition of complex solution from the previous part, with a concentration range of 1.2 to 5.4 × 10^−5^ M. The excitation wavelength was set to 520 nm, corresponding to the CT-DNA-EB complex, and the emission was followed between 540 and 650 nm. Both slits were set to 10 nm. The dependence of the relative fluorescence emission intensity decrease on the concentration of added complex was examined by Equation (1), while the thermodynamic parameters of binding were calculated for measurements at three temperatures (27, 35, and 37 °C) by Equation (2).

### 3.6. Molecular Docking

The optimized Fe(III)-PLITSC structure, obtained at the B3LYP/6–311++G (d,p) level of theory for all atoms except the Fe atom, and the def-TZVP basis set was used for Fe, served as the ligand in the molecular docking investigations. HSA (PDB ID: 4Z69) [54], BSA (PDB ID: 4F5S) [55], and DNA (PDB ID: 1BNA) [56] were selected as the target macromolecules. Docking calculations were executed using AutoDock4.2 [57], encompassing the entire volume of the target molecules to explore the optimal binding positions of the Fe(III)-PLITSC complex, with particular emphasis on regions surrounding Trp134 and Trp214.

## 4. Conclusions

A bis-ligand-iron(III) complex with differently protonated pyridoxal–*S*-methyl-isothiosemicarbazone ligands was obtained, and its crystal structure was solved. The deviation from the octahedral geometry occurred due to the protonation of one ligand. The latter interactions were formed with co-crystallized solvent molecules and counterions. The structure optimization at B3LYP/6-311++G(d,p)(H,C,N,O,S)/def2-TZVP(Fe) led to the high correlation coefficients and low MAE values between experimental and theoretical bond lengths and angles. The electron densities and Laplacians of the bond critical points outlined the effect of protonation on interactions between donor atoms and central metal ions. These differences were the most prominent in the case of hydrazine nitrogen. The binding affinity of the obtained complex towards BSA was between −31.0 and −34.9 kJ mol^−1^, while for HSA, it was between −33.0 and −36.4 kJ mol^−1^. The values obtained by molecular docking simulation were −28.8 (BSA) and −30.3 kJ mol^−1^ (HSA). These simulations proved that the investigated compound binds to the active positions of proteins, which leads to a decrease in fluorescence intensity. The binding affinity towards DNA was around −25.0 kJ mol^−1^ in the investigated temperature range. The calculated binding energy was −27.6 kJ mol^−1^ through intercalation within the minor groove. Based on these results, further experimental studies are advised to elucidate the biological effects in vitro and in vivo, especially cytotoxicity and mechanism of cell death analyses towards common cancer cell types.

## Figures and Tables

**Table 1:** The electron density and Laplacian of the most important stabilization interactions within the structure of [Fe(PLITSC–H)(PLITSC)]^2+^.

Ligand	Stabilization Interaction	Distance [Å]	Electron Density [a.u.]	Laplacian [a.u.]
**PLITSC**	Fe–O	1.92	0.090	0.521
	Fe–N_hydrazine_	1.96	0.091	0.528
	Fe–N_amino_	1.98	0.093	0.391
	N–N_hydrazine_	1.37	0.350	−0.648
	C=N_hydrazine_	1.38	0.309	−0.873
**PLITSC–H**	Fe–O	1.93	0.090	0.513
	Fe–N_hydrazine_	1.94	0.104	0.488
	Fe–N_amino_	1.89	0.119	0.440
	N–N_hydrazine_	1.37	0.356	−0.642
	C=N_hydrazine_	1.33	0.357	−1.131

**Table 2:** Binding process’s parameters for the interaction between obtained complexes and BSA.

Compound	T [K]	*K_b_* [M^−1^]	*n*	*R* ^2^	Δ*H_b_* [kJ mol^−1^]	Δ*S_b_* [J mol^−1^ K^−1^]	Δ*G_b_* [kJ mol^−1^]
**[Fe(PLITSC–H)(PLITSC)]^2+^**	300	2.43 × 10^5^	1.09	0.985	85.7	389.1	−31.0
	305	4.68 × 10^5^	1.14	0.998			−33.0
	310	7.35 × 10^5^	1.17	0.999			−34.9

**Table 3:** Binding process’s parameters for the interaction between obtained complexes and HSA.

Compound	T [K]	*K_b_* [M^−1^]	*n*	*R* ^2^	Δ*H_b_* [kJ mol^−1^]	Δ*S_b_* [J mol^−1^ K^−1^]	Δ*G_b_* [kJ mol^−1^]
**[Fe(PLITSC–H)(PLITSC)]^2+^**	300	6.08 × 10^5^	1.16	0.997	68.9	339.8	−33.0
	305	7.69 × 10^5^	1.19	0.982			−34.7
	310	1.49 × 10^6^	1.23	0.999			−36.4

**Table 4:** Binding process parameters for the interaction between the obtained complexes and DNA.

Compound	T [K]	*K_b_* [M^−1^]	*n*	*R* ^2^	Δ*H_b_* [kJ mol^−1^]	Δ*S_b_* [J mol^−1^ K^−1^]	Δ*G_b_* [kJ mol^−1^]
**[Fe(PLITSC–H)(PLITSC)]^2+^**	300	4.37 × 10^4^	1.16	0.999	−78.7	−173.4	−26.7
	308	2.00 × 10^4^	1.15	0.999			−25.3
	310	1.55 × 10^4^	1.12	0.999			−24.9

**Table 5:** Crystal data of compound ([Fe(PLITSC–H)(PLITSC)][SO_4_])_2_ ^.^ 5H_2_O.

Empirical formula	C_40_H_64_Fe_2_N_16_O_21_S_6_
Formula weight	1409.13
Temperature (K)	123 (2)
Wavelength (Å)	1.54184
Crystal System	monoclinic
Space group	*Cc*
Volume (Å^3^)	5834.2(2)
Unit cell dimension (Å/°)	*a* = 30.0490(6) *b* = 9.7008(2) *c* = 21.0593(4) *β* = 108.125(2)
Z	4

## Data Availability

Data are contained within this article.

## Supplementary Materials

The following supporting information can be downloaded at: https://www.mdpi.com/article/10.3390/ijms25137058/s1.
