# Supplementary material for: Crystal Structure, Theoretical Analysis, and Protein/DNA Binding Activity of Iron(III) Complex Containing Differently Protonated Pyridoxal–S-Methyl-Isothiosemicarbazone Ligands
Source: Int J Mol Sci. 2024 Jun 27;25(13):7058. doi: 10.3390/ijms25137058 (PMC11241004; doi:10.3390/ijms25137058)
Supplement: Supplementary file 1 [file ijms-25-07058-s001.zip › ijms-3032947-supplementary.pdf]

Supplementary informatio for:

Crystal Structure, Theoretical Analysis, and Protein/DNA Binding Activity of Iron(III) Complex Containing Differently Protonated Pyridoxal–*S*-Methyl-Isothiosemicarbazone Ligands

Violeta Jevtovic [1,*], Luka Golubović [2], Badriah Alshammari [1], Maha Raghyan Alshammari [1], Sahar Y. Rajeh [1], Maha Awjan Alreshidi [1], Odeh A.O. Alshammari [1], Aleksandra Rakić [2] and Dušan Dimić [2,*]

[1]Department of Chemistry, College of Science, University Ha'il, Ha'il 81451, Saudi Arabia

[2]Faculty of Physical Chemistry, University of Belgrade, Studentski trg 12-16, 11000 Belgrade, Serbia

* Correspondence: v.jevtovic@uoh.edu.sa (V.J.); ddimic@ffh.bg.ac.rs (D.D.)

Table S1. Atomic coordinates (x 10^4) and equivalent isotropic displacement parameters (Å^2 x 10^3) for obtained complex.

	x	y	z	U(eq)
Fe(1)	4495(1)	9771(1)	3415(1)	15(1)
S(1)	4185(1)	14311(2)	2787(1)	27(1)
O(1)	4304(1)	8602(4)	4040(2)	19(1)
N(1)	3470(2)	6338(5)	4335(3)	22(1)
C(1)	3893(2)	8132(6)	4020(3)	17(1)
Fe(2)	5510(1)	-66(1)	6644(1)	15(1)
S(2)	4016(1)	7081(2)	1558(1)	25(1)
O(2)	2201(2)	8229(5)	3568(3)	31(1)
N(2)	3823(2)	10758(5)	3369(2)	16(1)
C(2)	3881(2)	6865(6)	4343(3)	20(1)
S(3)	5835(1)	4530(2)	7179(1)	28(1)
O(3)	5065(1)	10265(4)	4135(2)	20(1)
N(3)	3769(2)	12091(5)	3131(2)	18(1)
C(3)	3056(2)	6959(6)	4043(3)	23(1)
S(4)	5978(1)	-2802(2)	8485(1)	22(1)
O(4)	6321(2)	6367(5)	4085(3)	42(1)
N(4)	4511(2)	11771(5)	3090(2)	20(1)
C(4)	3037(2)	8209(6)	3723(3)	19(1)
S(5)	2861(1)	3238(1)	4355(1)	24(1)
O(5)	5701(1)	-1251(4)	6023(2)	19(1)
N(5)	6279(2)	10622(5)	4943(2)	21(1)
C(5)	3460(2)	8815(6)	3705(3)	16(1)
S(6)	7056(1)	-6811(1)	5945(1)	20(1)
O(6)	7818(2)	-1911(5)	6728(3)	31(1)
N(6)	4974(2)	8387(5)	3142(2)	16(1)
C(6)	3447(2)	10173(6)	3415(3)	17(1)
O(7)	4943(1)	441(4)	5921(2)	19(1)
N(7)	4778(2)	7561(5)	2584(2)	18(1)
C(7)	4160(2)	12584(6)	3010(3)	18(1)
O(8)	3695(2)	-3502(5)	5930(3)	32(1)
N(8)	4125(2)	8898(5)	2542(2)	19(1)

C(8)	4322(2)	6095(7)	4687(3)	29(1)
O(9)	3340(2)	3769(5)	4680(3)	32(1)
N(9)	6528(2)	-3618(5)	5793(2)	21(1)
C(9)	2565(2)	8885(6)	3386(3)	24(1)
O(10)	2890(2)	1737(5)	4292(2)	32(1)
N(10)	6195(2)	878(5)	6698(2)	16(1)
C(10)	3629(3)	15010(7)	2770(4)	36(2)
O(11)	2565(2)	3553(6)	4770(3)	43(1)
N(11)	6246(2)	2237(5)	6902(2)	17(1)
C(11)	5507(2)	10006(5)	4285(3)	16(1)
O(12)	2662(2)	3882(5)	3700(2)	37(1)
N(12)	5509(2)	1973(5)	6953(2)	20(1)
C(12)	5820(2)	10844(6)	4788(3)	20(1)
O(13)	6610(1)	-6257(4)	5482(2)	27(1)
N(13)	3729(2)	742(5)	5081(2)	21(1)
C(13)	6476(2)	9625(6)	4662(3)	22(1)
O(14)	7452(1)	-6030(4)	5859(2)	23(1)
N(14)	5033(2)	-1467(5)	6904(2)	17(1)
C(14)	6195(2)	8781(6)	4187(3)	21(1)
O(15)	7051(1)	-6682(4)	6645(2)	26(1)
N(15)	5224(2)	-2313(5)	7451(2)	19(1)
C(15)	5705(2)	8966(5)	3985(3)	17(1)
O(16)	7089(1)	-8289(4)	5789(2)	22(1)
N(16)	5875(2)	-934(5)	7514(2)	22(1)
C(16)	5412(2)	8128(6)	3445(3)	18(1)
O(17)	2198(2)	10086(5)	4554(3)	34(1)
C(17)	4334(2)	7939(6)	2298(3)	20(1)
O(18)	6885(2)	-534(6)	8135(3)	35(1)
C(18)	5640(2)	11954(6)	5122(3)	21(1)
O(19)	2836(2)	2787(5)	2616(2)	34(1)
C(19)	6427(2)	7693(7)	3888(3)	27(1)
O(20)	7884(2)	99(5)	5853(3)	33(1)
C(20)	4444(3)	5953(7)	1404(3)	31(1)
O(21)	8119(2)	-4639(5)	6953(3)	33(1)
C(21)	6109(2)	-1767(6)	6070(3)	19(1)
C(22)	6117(2)	-3041(6)	5747(3)	19(1)
C(23)	6949(2)	-3050(6)	6137(3)	24(1)
C(24)	6968(2)	-1822(6)	6448(3)	21(1)
C(25)	6545(2)	-1142(6)	6412(3)	18(1)
C(26)	6565(2)	245(6)	6680(3)	17(1)
C(27)	5856(2)	2776(6)	7000(3)	18(1)
C(28)	5680(2)	-3763(7)	5364(3)	26(1)
C(29)	7439(2)	-1239(6)	6869(3)	26(1)
C(30)	6437(3)	5078(7)	7400(4)	37(2)
C(31)	4499(2)	154(6)	5751(3)	18(1)
C(32)	4189(2)	969(6)	5238(3)	19(1)
C(33)	3538(2)	-234(7)	5363(3)	24(1)
C(34)	3810(2)	-1094(6)	5843(3)	21(1)
C(35)	4303(2)	-895(6)	6048(3)	17(1)
C(36)	4594(2)	-1716(6)	6593(3)	18(1)
C(37)	5663(2)	-1932(6)	7746(3)	18(1)
C(38)	4366(2)	2070(6)	4881(3)	22(1)
C(39)	3579(2)	-2198(7)	6130(3)	28(1)
C(40)	5548(2)	-3997(7)	8602(3)	29(1)

U(eq) is defined as one third of the trace of the orthogonalized Uij tensor.

Table S2. Bond lengths [Å] and angles [°] for obtained complex.

Fe(1)-O(1)	1.953(4)
Fe(1)-O(3)	1.962(4)
Fe(1)-N(8)	2.015(5)
Fe(1)-N(4)	2.063(5)
Fe(1)-N(6)	2.173(4)
Fe(1)-N(2)	2.211(5)
S(1)-C(7)	1.748(6)
S(1)-C(10)	1.791(8)
O(1)-C(1)	1.305(6)
N(1)-C(2)	1.333(8)
N(1)-C(3)	1.345(8)
C(1)-C(2)	1.411(8)
C(1)-C(5)	1.428(7)
Fe(2)-O(5)	1.956(4)
Fe(2)-O(7)	1.961(4)
Fe(2)-N(16)	2.006(5)
Fe(2)-N(12)	2.083(5)
Fe(2)-N(14)	2.164(5)
Fe(2)-N(10)	2.224(5)
S(2)-C(17)	1.762(6)
S(2)-C(20)	1.794(7)
O(2)-C(9)	1.416(7)
N(2)-C(6)	1.292(7)
N(2)-N(3)	1.378(6)
C(2)-C(8)	1.496(8)
S(3)-C(27)	1.748(5)
S(3)-C(30)	1.802(8)
O(3)-C(11)	1.291(7)
N(3)-C(7)	1.364(7)
C(3)-C(4)	1.381(8)
S(4)-C(37)	1.765(6)
S(4)-C(40)	1.808(7)
O(4)-C(19)	1.418(9)
N(4)-C(7)	1.284(8)
C(4)-C(5)	1.411(7)
C(4)-C(9)	1.523(8)
S(5)-O(11)	1.462(5)
S(5)-O(12)	1.463(5)
S(5)-O(10)	1.467(5)
S(5)-O(9)	1.479(5)
O(5)-C(21)	1.299(7)
N(5)-C(12)	1.333(8)
N(5)-C(13)	1.361(8)
C(5)-C(6)	1.448(8)
S(6)-O(14)	1.468(4)
S(6)-O(16)	1.481(4)

S(6)-O(15)	1.486(4)
S(6)-O(13)	1.491(4)
O(6)-C(29)	1.423(7)
N(6)-C(16)	1.296(7)
N(6)-N(7)	1.392(6)
O(7)-C(31)	1.300(7)
N(7)-C(17)	1.332(8)
O(8)-C(39)	1.411(8)
N(8)-C(17)	1.315(8)
N(9)-C(22)	1.330(7)
N(9)-C(23)	1.365(8)
N(10)-C(26)	1.281(7)
N(10)-N(11)	1.381(6)
N(11)-C(27)	1.355(7)
C(11)-C(15)	1.415(8)
C(11)-C(12)	1.430(8)
N(12)-C(27)	1.282(7)
C(12)-C(18)	1.477(8)
N(13)-C(32)	1.336(8)
N(13)-C(33)	1.340(8)
C(13)-C(14)	1.363(8)
N(14)-C(36)	1.302(7)
N(14)-N(15)	1.385(6)
C(14)-C(15)	1.412(8)
C(14)-C(19)	1.506(8)
N(15)-C(37)	1.323(7)
C(15)-C(16)	1.451(8)
N(16)-C(37)	1.333(7)
C(21)-C(22)	1.415(8)
C(21)-C(25)	1.420(8)
C(22)-C(28)	1.486(8)
C(23)-C(24)	1.352(8)
C(24)-C(25)	1.412(8)
C(24)-C(29)	1.526(8)
C(25)-C(26)	1.453(7)
C(31)-C(35)	1.415(8)
C(31)-C(32)	1.426(8)
C(32)-C(38)	1.495(8)
C(33)-C(34)	1.368(9)
C(34)-C(35)	1.421(8)
C(34)-C(39)	1.502(9)
C(35)-C(36)	1.445(8)
O(1)-Fe(1)-O(3)	89.43(17)
O(1)-Fe(1)-N(8)	100.17(19)
O(3)-Fe(1)-N(8)	155.42(18)
O(1)-Fe(1)-N(4)	144.20(18)
O(3)-Fe(1)-N(4)	85.51(19)
N(8)-Fe(1)-N(4)	98.9(2)
O(1)-Fe(1)-N(6)	100.21(17)
O(3)-Fe(1)-N(6)	82.18(17)
N(8)-Fe(1)-N(6)	73.90(18)
N(4)-Fe(1)-N(6)	114.09(18)
O(1)-Fe(1)-N(2)	79.93(17)

O(3)-Fe(1)-N(2)	119.63(17)
N(8)-Fe(1)-N(2)	84.51(19)
N(4)-Fe(1)-N(2)	72.10(18)
N(6)-Fe(1)-N(2)	158.14(18)
C(7)-S(1)-C(10)	104.5(3)
C(1)-O(1)-Fe(1)	130.8(4)
C(2)-N(1)-C(3)	123.8(5)
O(1)-C(1)-C(2)	117.1(5)
O(1)-C(1)-C(5)	124.8(5)
C(2)-C(1)-C(5)	118.2(5)
O(5)-Fe(2)-O(7)	89.66(17)
O(5)-Fe(2)-N(16)	99.94(19)
O(7)-Fe(2)-N(16)	155.65(18)
O(5)-Fe(2)-N(12)	142.74(18)
O(7)-Fe(2)-N(12)	85.38(19)
N(16)-Fe(2)-N(12)	99.6(2)
O(5)-Fe(2)-N(14)	98.94(17)
O(7)-Fe(2)-N(14)	82.60(17)
N(16)-Fe(2)-N(14)	73.86(19)
N(12)-Fe(2)-N(14)	116.89(18)
O(5)-Fe(2)-N(10)	79.42(17)
O(7)-Fe(2)-N(10)	120.21(17)
N(16)-Fe(2)-N(10)	83.73(19)
N(12)-Fe(2)-N(10)	71.54(18)
N(14)-Fe(2)-N(10)	156.97(18)
C(17)-S(2)-C(20)	103.0(3)
C(6)-N(2)-N(3)	115.7(5)
C(6)-N(2)-Fe(1)	127.8(4)
N(3)-N(2)-Fe(1)	115.1(3)
N(1)-C(2)-C(1)	119.3(5)
N(1)-C(2)-C(8)	119.6(5)
C(1)-C(2)-C(8)	121.1(5)
C(27)-S(3)-C(30)	103.9(3)
C(11)-O(3)-Fe(1)	136.6(4)
C(7)-N(3)-N(2)	112.8(4)
N(1)-C(3)-C(4)	120.5(5)
C(37)-S(4)-C(40)	102.5(3)
C(7)-N(4)-Fe(1)	121.3(4)
C(3)-C(4)-C(5)	118.6(5)
C(3)-C(4)-C(9)	119.8(5)
C(5)-C(4)-C(9)	121.7(5)
O(11)-S(5)-O(12)	109.1(3)
O(11)-S(5)-O(10)	109.0(3)
O(12)-S(5)-O(10)	110.9(3)
O(11)-S(5)-O(9)	109.7(3)
O(12)-S(5)-O(9)	110.0(3)
O(10)-S(5)-O(9)	108.2(3)
C(21)-O(5)-Fe(2)	130.0(4)
C(12)-N(5)-C(13)	124.4(5)
C(4)-C(5)-C(1)	119.5(5)
C(4)-C(5)-C(6)	119.0(5)
C(1)-C(5)-C(6)	121.2(5)
O(14)-S(6)-O(16)	111.1(2)
O(14)-S(6)-O(15)	109.7(2)

O(16)-S(6)-O(15)	108.8(2)
O(14)-S(6)-O(13)	109.4(2)
O(16)-S(6)-O(13)	108.2(2)
O(15)-S(6)-O(13)	109.5(2)
C(16)-N(6)-N(7)	113.8(4)
C(16)-N(6)-Fe(1)	130.1(4)
N(7)-N(6)-Fe(1)	115.9(3)
N(2)-C(6)-C(5)	121.5(5)
C(31)-O(7)-Fe(2)	136.8(4)
C(17)-N(7)-N(6)	109.0(4)
N(4)-C(7)-N(3)	118.6(5)
N(4)-C(7)-S(1)	121.5(4)
N(3)-C(7)-S(1)	119.8(4)
C(17)-N(8)-Fe(1)	117.3(4)
C(22)-N(9)-C(23)	123.8(5)
O(2)-C(9)-C(4)	111.5(5)
C(26)-N(10)-N(11)	116.9(5)
C(26)-N(10)-Fe(2)	126.8(4)
N(11)-N(10)-Fe(2)	114.9(3)
C(27)-N(11)-N(10)	113.4(4)
O(3)-C(11)-C(15)	124.9(5)
O(3)-C(11)-C(12)	117.3(5)
C(15)-C(11)-C(12)	117.8(5)
C(27)-N(12)-Fe(2)	121.4(4)
N(5)-C(12)-C(11)	118.7(5)
N(5)-C(12)-C(18)	120.3(5)
C(11)-C(12)-C(18)	121.0(5)
C(32)-N(13)-C(33)	124.2(5)
N(5)-C(13)-C(14)	119.5(5)
C(36)-N(14)-N(15)	113.7(5)
C(36)-N(14)-Fe(2)	129.5(4)
N(15)-N(14)-Fe(2)	116.6(3)
C(13)-C(14)-C(15)	119.6(5)
C(13)-C(14)-C(19)	117.8(5)
C(15)-C(14)-C(19)	122.6(5)
C(37)-N(15)-N(14)	108.6(4)
C(14)-C(15)-C(11)	120.0(5)
C(14)-C(15)-C(16)	119.1(5)
C(11)-C(15)-C(16)	120.9(5)
C(37)-N(16)-Fe(2)	116.8(4)
N(6)-C(16)-C(15)	123.6(5)
N(8)-C(17)-N(7)	123.4(5)
N(8)-C(17)-S(2)	119.0(4)
N(7)-C(17)-S(2)	117.5(4)
O(4)-C(19)-C(14)	109.7(5)
O(5)-C(21)-C(22)	117.2(5)
O(5)-C(21)-C(25)	125.0(5)
C(22)-C(21)-C(25)	117.7(5)
N(9)-C(22)-C(21)	119.2(5)
N(9)-C(22)-C(28)	119.0(5)
C(21)-C(22)-C(28)	121.8(5)
C(24)-C(23)-N(9)	120.2(5)
C(23)-C(24)-C(25)	119.0(5)
C(23)-C(24)-C(29)	119.9(5)

C(25)-C(24)-C(29)	120.9(5)
C(24)-C(25)-C(21)	120.0(5)
C(24)-C(25)-C(26)	118.9(5)
C(21)-C(25)-C(26)	120.8(5)
N(10)-C(26)-C(25)	121.6(5)
N(12)-C(27)-N(11)	118.4(5)
N(12)-C(27)-S(3)	121.6(4)
N(11)-C(27)-S(3)	120.0(4)
O(6)-C(29)-C(24)	111.5(5)
O(7)-C(31)-C(35)	124.4(5)
O(7)-C(31)-C(32)	117.4(5)
C(35)-C(31)-C(32)	118.2(5)
N(13)-C(32)-C(31)	118.4(5)
N(13)-C(32)-C(38)	119.6(5)
C(31)-C(32)-C(38)	121.9(5)
N(13)-C(33)-C(34)	121.2(5)
C(33)-C(34)-C(35)	118.0(5)
C(33)-C(34)-C(39)	119.1(6)
C(35)-C(34)-C(39)	122.9(5)
C(31)-C(35)-C(34)	120.0(5)
C(31)-C(35)-C(36)	121.0(5)
C(34)-C(35)-C(36)	118.8(5)
N(14)-C(36)-C(35)	124.4(5)
N(15)-C(37)-N(16)	123.6(5)
N(15)-C(37)-S(4)	117.6(4)
N(16)-C(37)-S(4)	118.9(4)
O(8)-C(39)-C(34)	109.3(5)

Symmetry transformations used to generate equivalent atoms:

Table S3. Anisotropic displacement parameters ($Å^2 \times 10^3$) for obtained complex.

	U11	U22	U33	U23	U13	U12
Fe(1)	11(1)	15(1)	17(1)	0(1)	4(1)	0(1)
S(1)	28(1)	18(1)	38(1)	9(1)	16(1)	1(1)
O(1)	12(2)	23(2)	22(2)	7(2)	3(2)	0(1)
N(1)	22(3)	19(2)	29(2)	5(2)	11(2)	-1(2)
C(1)	13(2)	20(2)	17(2)	1(2)	4(2)	0(2)
Fe(2)	12(1)	16(1)	18(1)	1(1)	5(1)	0(1)
S(2)	25(1)	28(1)	19(1)	-4(1)	4(1)	-10(1)
O(2)	16(2)	23(2)	56(3)	5(2)	13(2)	-2(2)
N(2)	17(2)	15(2)	18(2)	0(2)	6(2)	1(2)
C(2)	20(3)	19(3)	24(3)	2(2)	8(2)	2(2)
S(3)	27(1)	19(1)	42(1)	-10(1)	18(1)	-1(1)
O(3)	14(2)	23(2)	21(2)	-5(2)	4(2)	-2(2)
N(3)	20(2)	13(2)	25(2)	6(2)	10(2)	3(2)
C(3)	19(3)	20(3)	30(3)	2(2)	9(2)	-2(2)
S(4)	22(1)	24(1)	19(1)	4(1)	5(1)	7(1)
O(4)	22(2)	24(2)	78(4)	-9(2)	11(2)	0(2)

N(4)	16(2)	20(2)	24(2)	3(2)	7(2)	-1(2)
C(4)	14(2)	18(2)	26(3)	0(2)	8(2)	0(2)
S(5)	21(1)	17(1)	31(1)	2(1)	4(1)	2(1)
O(5)	14(2)	18(2)	25(2)	-3(2)	6(2)	1(1)
N(5)	18(2)	24(2)	20(2)	-1(2)	3(2)	-8(2)
C(5)	12(2)	17(2)	19(2)	0(2)	4(2)	2(2)
S(6)	16(1)	16(1)	26(1)	-5(1)	5(1)	-2(1)
O(6)	18(2)	24(2)	52(3)	-5(2)	14(2)	2(2)
N(6)	17(2)	14(2)	17(2)	-4(2)	7(2)	-4(2)
C(6)	14(3)	19(3)	17(2)	2(2)	5(2)	1(2)
O(7)	11(2)	22(2)	22(2)	3(2)	3(2)	0(1)
N(7)	13(2)	21(2)	18(2)	-4(2)	5(2)	-4(2)
C(7)	16(3)	17(2)	18(2)	4(2)	2(2)	-1(2)
O(8)	23(2)	26(2)	50(3)	1(2)	14(2)	2(2)
N(8)	12(2)	21(2)	20(2)	-1(2)	-1(2)	-2(2)
C(8)	18(3)	28(3)	38(3)	16(3)	4(3)	7(2)
O(9)	21(2)	24(2)	49(3)	17(2)	7(2)	-1(2)
N(9)	16(2)	18(2)	27(2)	-4(2)	5(2)	3(2)
C(9)	14(3)	18(3)	41(3)	6(2)	7(2)	0(2)
O(10)	37(3)	18(2)	35(2)	1(2)	1(2)	5(2)
N(10)	15(2)	15(2)	21(2)	-1(2)	8(2)	0(2)
C(10)	41(4)	22(3)	50(4)	6(3)	23(3)	5(3)
O(11)	27(2)	61(3)	42(3)	-15(2)	12(2)	-4(2)
N(11)	13(2)	14(2)	25(2)	-3(2)	8(2)	-2(2)
C(11)	13(3)	17(2)	14(3)	2(2)	0(2)	-5(2)
O(12)	39(2)	31(2)	39(2)	8(2)	7(2)	12(2)
N(12)	17(2)	21(2)	24(2)	-3(2)	8(2)	1(2)
C(12)	19(3)	20(3)	20(3)	4(2)	7(2)	0(2)
O(13)	16(2)	22(2)	38(2)	-7(2)	2(2)	1(2)
N(13)	14(2)	29(3)	19(2)	2(2)	3(2)	7(2)
C(13)	15(3)	22(3)	27(3)	0(2)	5(2)	-1(2)
O(14)	16(2)	24(2)	28(2)	-2(2)	6(1)	-6(1)
N(14)	16(2)	16(2)	20(2)	2(2)	5(2)	2(2)
C(14)	12(3)	26(3)	22(3)	1(2)	3(2)	1(2)
O(15)	23(2)	29(2)	31(2)	-7(2)	14(2)	-4(2)
N(15)	21(2)	18(2)	20(2)	4(2)	10(2)	4(2)
C(15)	17(3)	18(2)	17(2)	2(2)	5(2)	0(2)
O(16)	20(2)	20(2)	26(2)	-5(2)	5(2)	-1(2)
N(16)	18(2)	25(2)	22(2)	4(2)	5(2)	0(2)
C(16)	16(2)	17(2)	20(2)	-1(2)	7(2)	1(2)
O(17)	30(2)	32(2)	44(3)	10(2)	16(2)	1(2)
C(17)	21(3)	18(3)	19(2)	0(2)	7(2)	-8(2)
O(18)	22(2)	30(3)	44(3)	0(2)	-3(2)	-4(2)
C(18)	21(3)	21(3)	20(3)	-2(2)	6(2)	-5(2)
O(19)	24(2)	39(3)	36(2)	0(2)	4(2)	4(2)
C(19)	15(3)	32(3)	33(3)	-9(2)	6(2)	1(2)
O(20)	28(2)	29(2)	46(3)	-3(2)	16(2)	0(2)
C(20)	37(4)	35(3)	25(3)	-11(3)	16(3)	-12(3)
O(21)	24(2)	28(3)	36(2)	0(2)	-5(2)	-1(2)
C(21)	22(3)	18(2)	18(2)	1(2)	9(2)	2(2)
C(22)	14(3)	19(3)	22(3)	0(2)	3(2)	3(2)
C(23)	15(3)	21(3)	35(3)	-1(2)	6(2)	5(2)
C(24)	21(3)	17(3)	28(3)	0(2)	10(2)	0(2)
C(25)	21(3)	14(2)	22(3)	2(2)	9(2)	0(2)

C(26)	14(3)	15(2)	22(3)	2(2)	6(2)	-1(2)
C(27)	23(3)	16(2)	17(2)	-3(2)	8(2)	2(2)
C(28)	23(3)	23(3)	32(3)	-9(2)	6(2)	-2(2)
C(29)	14(2)	18(3)	46(3)	-3(2)	9(2)	1(2)
C(30)	36(4)	28(3)	55(4)	-19(3)	25(3)	-11(3)
C(31)	17(3)	19(3)	19(3)	-1(2)	7(2)	3(2)
C(32)	17(3)	22(3)	16(2)	-1(2)	2(2)	8(2)
C(33)	11(3)	36(3)	23(3)	-4(2)	4(2)	3(2)
C(34)	20(3)	25(3)	22(3)	-1(2)	11(2)	3(2)
C(35)	13(2)	18(2)	22(3)	-3(2)	7(2)	3(2)
C(36)	14(2)	17(2)	25(3)	0(2)	9(2)	-1(2)
C(37)	13(2)	18(2)	23(3)	-1(2)	6(2)	7(2)
C(38)	26(3)	18(3)	24(3)	1(2)	9(2)	4(2)
C(39)	20(3)	31(3)	33(3)	0(3)	8(2)	3(2)
C(40)	33(3)	29(3)	28(3)	8(3)	16(3)	8(3)

The anisotropic displacement factor exponent takes the form:
-2 pi^2 [h^2 a*^2 U11 + ... + 2 h k a* b* U12]

Table S4. Hydrogen coordinates (x 10^4) and isotropic displacement parameters (Å^2 x 10^3) for obtained complexes.

	x	y	z	U(eq)
H(3)	2775	6533	4058	27
H(6)	3157	10645	3255	20
H(8A)	4249	5300	4924	43
H(8B)	4540	6705	5007	43
H(8C)	4466	5774	4355	43
H(9A)	2579	9869	3515	29
H(9B)	2495	8838	2895	29
H(10A)	3381	14586	2404	53
H(10B)	3630	16009	2701	53
H(10C)	3573	14816	3196	53
H(13)	6806	9519	4796	26
H(16)	5549	7347	3307	21
H(18A)	5903	12504	5398	31
H(18B)	5470	11549	5405	31
H(18C)	5429	12548	4784	31
H(19A)	6315	7766	3395	33
H(19B)	6771	7835	4041	33
H(20A)	4684	6501	1294	46
H(20B)	4292	5340	1030	46
H(20C)	4591	5401	1805	46
H(23)	7231	-3519	6158	29
H(26)	6860	692	6847	20
H(28A)	5759	-4640	5194	40
H(28B)	5504	-3185	4987	40
H(28C)	5488	-3938	5656	40
H(29A)	7449	-240	6779	31

H(29B)	7471	-1353	7349	31
H(30A)	6625	4545	7785	56
H(30B)	6458	6060	7514	56
H(30C)	6555	4926	7021	56
H(33)	3207	-329	5229	28
H(36)	4454	-2490	6733	22
H(38A)	4100	2552	4571	33
H(38B)	4557	1652	4631	33
H(38C)	4556	2727	5207	33
H(39A)	3686	-2136	6623	33
H(39B)	3235	-2071	5970	33
H(40A)	5305	-3486	8722	43
H(40B)	5701	-4644	8961	43
H(40C)	5406	-4508	8187	43
H(4D)	6570(17)	5900(70)	4280(30)	27(19)
H(8D)	3470(30)	-4090(70)	5910(70)	120(60)
H(1N)	3440(40)	5560(70)	4560(50)	60(30)
H(3N)	3471(12)	12410(70)	3010(30)	23(17)
H(4N)	4750(30)	12250(110)	3020(60)	80(40)
H(5N)	6480(20)	11140(70)	5250(30)	29(19)
H(8N)	3817(10)	9030(60)	2330(30)	8(14)
H(9N)	6560(30)	-4490(40)	5660(40)	30(19)
H(11N)	6478(19)	2790(60)	6870(40)	29(19)
H(12N)	5245(14)	2310(60)	7020(30)	5(13)
H(13N)	3490(20)	1150(80)	4770(30)	27(19)
H(16N)	6179(11)	-760(80)	7730(30)	30(20)
H(2D)	2110(50)	8780(130)	3840(60)	110(50)
H(6D)	7850(30)	-1560(80)	6350(20)	26(19)
H(17D)	2250(30)	9760(90)	4960(20)	50(30)
H(17E)	2360(50)	10850(90)	4600(60)	120(60)
H(18D)	6950(40)	-1350(50)	8310(50)	80(40)
H(18E)	7130(30)	-50(90)	8350(60)	90(50)
H(19D)	2790(50)	3090(130)	2990(40)	120(50)
H(19E)	2620(30)	2130(100)	2480(60)	100(50)
H(20D)	7650(20)	710(70)	5840(40)	50(20)
H(20E)	7780(20)	-130(60)	5429(15)	9(15)
H(21A)	8000(20)	-3810(40)	6880(40)	29(19)
H(21B)	7930(20)	-5200(60)	6680(30)	16(16)

Table S5. Hydrogen bonds for obtained complex [Å and °].

D-H...A	d(D-H)	d(H...A)	d(D...A)	<(DHA)
O(4)-H(4D)...O(17)#1	0.86(3)	1.96(3)	2.801(7)	165(7)
O(8)-H(8D)...O(20)#2	0.88(3)	1.89(4)	2.749(7)	165(8)
N(1)-H(1N)...O(9)	0.91(3)	1.79(5)	2.659(7)	158(10)
N(3)-H(3N)...O(19)#3	0.91(3)	1.86(3)	2.757(7)	168(7)
N(4)-H(4N)...N(15)#4	0.90(3)	2.14(8)	2.912(6)	144(11)
N(5)-H(5N)...O(16)#5	0.90(3)	1.91(4)	2.742(6)	155(8)
N(8)-H(8N)...O(21)#6	0.90(2)	2.08(3)	2.975(7)	170(6)
N(9)-H(9N)...O(13)	0.90(3)	1.78(3)	2.674(7)	174(8)
N(11)-H(11N)...O(15)#3	0.90(3)	1.99(4)	2.838(6)	157(7)
N(12)-H(12N)...N(7)#7	0.90(2)	2.11(4)	2.934(6)	151(5)
N(13)-H(13N)...O(10)	0.90(3)	1.86(4)	2.730(7)	161(7)
N(16)-H(16N)...O(18)	0.90(3)	2.04(3)	2.931(7)	173(7)
O(2)-H(2D)...O(17)	0.88(3)	1.93(7)	2.751(8)	154(15)
O(6)-H(6D)...O(20)	0.89(3)	1.95(5)	2.733(7)	146(7)
O(17)-H(17D)...O(14)#8	0.88(3)	1.95(3)	2.830(7)	171(9)
O(17)-H(17E)...O(10)#3	0.88(3)	2.07(9)	2.813(7)	141(13)
O(18)-H(18D)...O(2)#9	0.87(3)	1.98(4)	2.834(7)	167(12)
O(18)-H(18E)...O(12)#9	0.88(3)	1.90(3)	2.776(7)	173(12)
O(19)-H(19D)...O(12)	0.89(3)	1.83(3)	2.714(7)	173(15)
O(19)-H(19E)...O(15)#10	0.88(3)	2.09(9)	2.809(6)	138(11)
O(20)-H(20D)...O(16)#3	0.90(3)	1.93(3)	2.824(6)	169(8)
O(20)-H(20E)...O(11)#1	0.88(2)	1.85(4)	2.647(7)	150(6)
O(21)-H(21A)...O(6)	0.87(3)	1.92(3)	2.790(7)	172(7)
O(21)-H(21B)...O(14)	0.87(3)	2.03(3)	2.876(6)	163(6)

Symmetry transformations used to generate equivalent atoms:
#1 x+1/2,y-1/2,z #2 x-1/2,y-1/2,z #3 x,y+1,z
#4 x,-y+1,z-1/2 #5 x,y+2,z #6 x-1/2,-y+1/2,z-1/2
#7 x,-y+1,z+1/2 #8 x-1/2,y+3/2,z #9 x+1/2,-y+1/2,z+1/2
#10 x-1/2,-y-1/2,z-1/2

Table S6. The experimental and theoretical (B3LYP/6-311++G(d,p)(H,C,N,O,S)/def2-TZVP(Fe) level of theory) bond lenghts of $[Fe(PLITSC{-}H)(PLITSC)]^{2+}$ (in Å)

	Optimized	Crystallographic
Fe1 - O3	1.923	1.953
Fe1 - N8	1.959	2.211
O3 - C5	1.289	1.305
C5 - C18	1.433	1.427
C18 - C20	1.448	1.447
C20 - N8	1.293	1.294

N8 - N11	1.367	1.378
N11 - C23	1.376	1.364
C23 - N15	1.293	1.284
C23 - S2	1.756	1.748
S2 - C32	1.825	1.792
C5 - C9	1.432	1.411
C9 - C25	1.489	1.497
C9 - N4	1.337	1.333
N4 - C12	1.358	1.345
C12 - C16	1.373	1.380
C16 - C29	1.517	1.524
C29 - O7	1.410	1.415
Fe1 - N24	1.890	2.015
Fe1 - N19	1.937	2.173
N19 - N22	1.361	1.393
N22 - C44	1.329	1.333
C44 - S6	1.752	1.763
S6 - C52	1.828	1.794
N19 - C42	1.302	1.296
C42 - C41	1.443	1.451
C41 - C36	1.433	1.416
C36 - O10	1.301	1.290
C36 - C37	1.419	1.431
C37 - C45	1.492	1.478
C37 - N17	1.347	1.332
N17 - C38	1.356	1.362
C38 - C40	1.374	1.363
C40 - C41	1.425	1.412
C40 - C49	1.521	1.506
C49 - O14	1.422	1.418
Fe1-O10	1.930	1.962
Fe1-N15	1.975	2.063

Table S7. The experimental and theoretical (B3LYP/6-311++G(d,p)(H,C,N,O,S)/def2-TZVP(Fe) level of theory) bond angles of $[Fe(PLITSC{-}H)(PLITSC)]^{2+}$ (in °)

	Optimized	Crystallographic
O3 - Fe1 - N8	91.89	79.94
Fe1 - N8 - N11	111.50	115.08
N8 - N11 - C23	116.61	112.79
N11 - C23 - N15	116.18	118.61
N15 - C23 - S2	129.75	121.52
C23 - S2 - C32	101.90	104.54

S2 - C23 - N11	114.07	119.81
Fe1 - N8 - C20	128.53	127.85
N8 - C20 - C18	123.53	121.42
C20 - C18 - C5	121.68	121.29
C18 - C5 - O3	126.82	124.78
Fe1 - O3 - C5	126.80	130.85
C20 - C18 - C16	118.52	118.96
C5 - C18 - C16	119.80	119.54
C18 - C16 - C29	122.70	121.62
C16 - C29 - O7	108.23	111.44
C29 - C16 - C12	117.93	119.78
C18 - C16 - C12	119.37	118.58
C16 - C12 - N4	119.29	120.58
C12 - N4 - C9	125.39	123.73
N4 - C9 - C25	120.04	119.51
C25 - C9 - C5	121.53	121.06
N4 - C9 - C5	118.43	119.42
C9 - C5 -C18	117.71	118.14
O3 - C5 - C9	115.47	117.08
N8 - Fe1 - N24	96.44	84.50
O3 - Fe1 - N24	92.12	100.16
N8 - Fe1 - N19	177.48	158.13
O3 - Fe1 - N19	88.07	100.20
N19 - Fe1 - N24	81.04	73.90
Fe1 - N24 - C44	112.32	117.27
N24 - C44 - S6	118.99	119.07
C44 - S6 - C52	103.02	103.03
S6 - C44 - N22	120.14	117.44
N24 - C44 - N22	120.87	123.48
C44 - N22 - N19	110.01	108.95
Fe1 - N19 - N22	115.71	115.91
Fe1 - N19 - C42	127.33	130.13
N22 - N19 - C42	116.83	113.75
N19 - C42 - C41	124.59	123.54
C42 - C41- C36	122.36	120.83
C42 - C41 - C40	118.45	119.10
C41 - C40 -C49	122.28	122.54
C40 - C49 - O14	112.78	109.73
C49 - C40 - C38	118.28	117.81
C41 - C40 - C38	119.43	119.64
C40 - C38 - N17	119.86	119.46
C38 - N17 - C37	124.33	124.43
N17 - C37 - C36	118.88	118.78
C37 - C36 - C41	118.32	117.68

C37 - C36 - O10	116.09	117.38
O10 - C36 - C41	125.59	124.93
N17 - C37 - C45	119.22	120.36
C45 - C37 - C36	121.90	120.84
O3-Fe1-N15	172.98	144.20
O3-Fe1-O10	90.60	89.41
N8-Fe1-N15	81.22	72.10
N8-Fe1-O10	89.55	119.64
N15-Fe1-N19	98.87	114.09
N15-Fe1-N24	89.99	98.95
N15-Fe1-O10	88.03	85.53
N19-Fe1-O10	92.97	82.19
N24-Fe1-O10	173.34	155.43

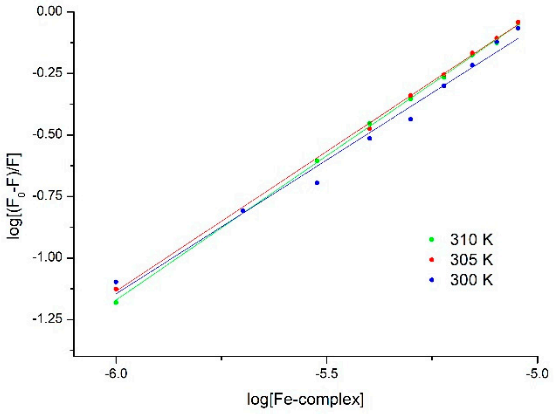

(a)

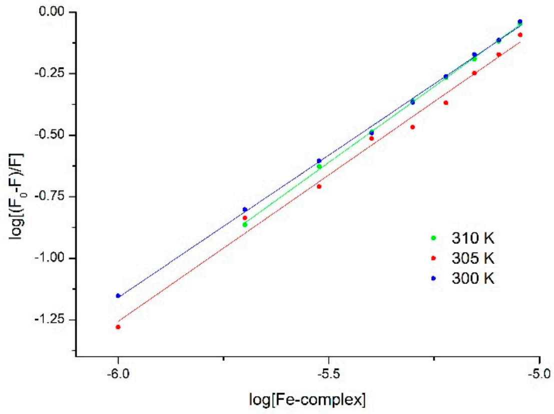

(b)

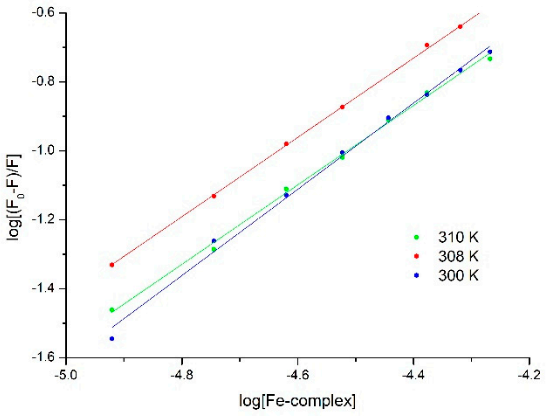

(c)

Figure S1. The dependency of the florescence emission decrease with increased concentration of quencher according to the Stern-Volmer equation for the binding of complex to (a) BSA, (b) HAS, and (c) CT-DNA.

Table S8. Molecular docking analysis of adducts formed by the $[Fe(PLITSC{-}H)(PLITSC)]^{2+}$ complex with HSA (Human Serum Albumin), BSA (Bovine Serum Albumin), and B-DNA.

Energy rank	HSA		BSA		B-DNA	
	ΔG [kJ mol^{-1}]	Subdomain (Binding site)	ΔG [kJ mol^{-1}]	Subdomain (Binding site)	ΔG [kJ mol^{-1}]	Side (Nucleic acids)
1.	-30.3	IIA (FA7) near Trp214	-28.8	IB (FA1) near Trp134	-27.6	Minor groove (ACTG)
2.	-25.9	IB (FA1)	-24.0	IIIA (outside)	-26.8	Minor groove (ACCG)
3.	-25.6	IB (FA1)	-18.5	IB (FA9)	-26.4	Minor groove (CTTT)
4.	-24.2	IB (FA1)	-17.8	IIA (FA8) near Trp213	-26.4	Minor groove (CTTT)
5.	-14.8	IIIA (FA3)	-0.8	IIB (FA6)	-25.5	Minor groove (AACCGG)
6.	+28.4	IA (FA2)	+155.7	IIIB (FA5)	-25.5	Minor groove (CCGG)
7.	+32.6	IA (FA2)	+157.8	IIIB (FA5)	-25.5	Minor groove (AACC)
8.	+54.9	IIIB (FA5)	+161.8	IIIB (FA5)	-25.5	Major groove (AAAATG)
9.	+58.6	IIIB (FA5)	+194.1	IIIB (FA5)	-25.1	Minor groove (AACTT)
10.	+94.4	IIIA (FA4)	+197.5	IIIB (FA5)		

Data concerning the adducts formed with BSA (Bovine Serum Albumin) and HSA (Human Serum Albumin) encompass the change in Gibbs free energy of binding (ΔG) within the subdomain and fatty acid binding site (FA) where the ligand $[Fe(PLITSC{-}1)(PLITSC)]^{2+}$ binds. Additionally, for adducts involving B-DNA, information regarding the orientation of the bound ligand (approaching either the minor groove or major groove side) and the specific nucleic acids with which the ligand interacts is provided.

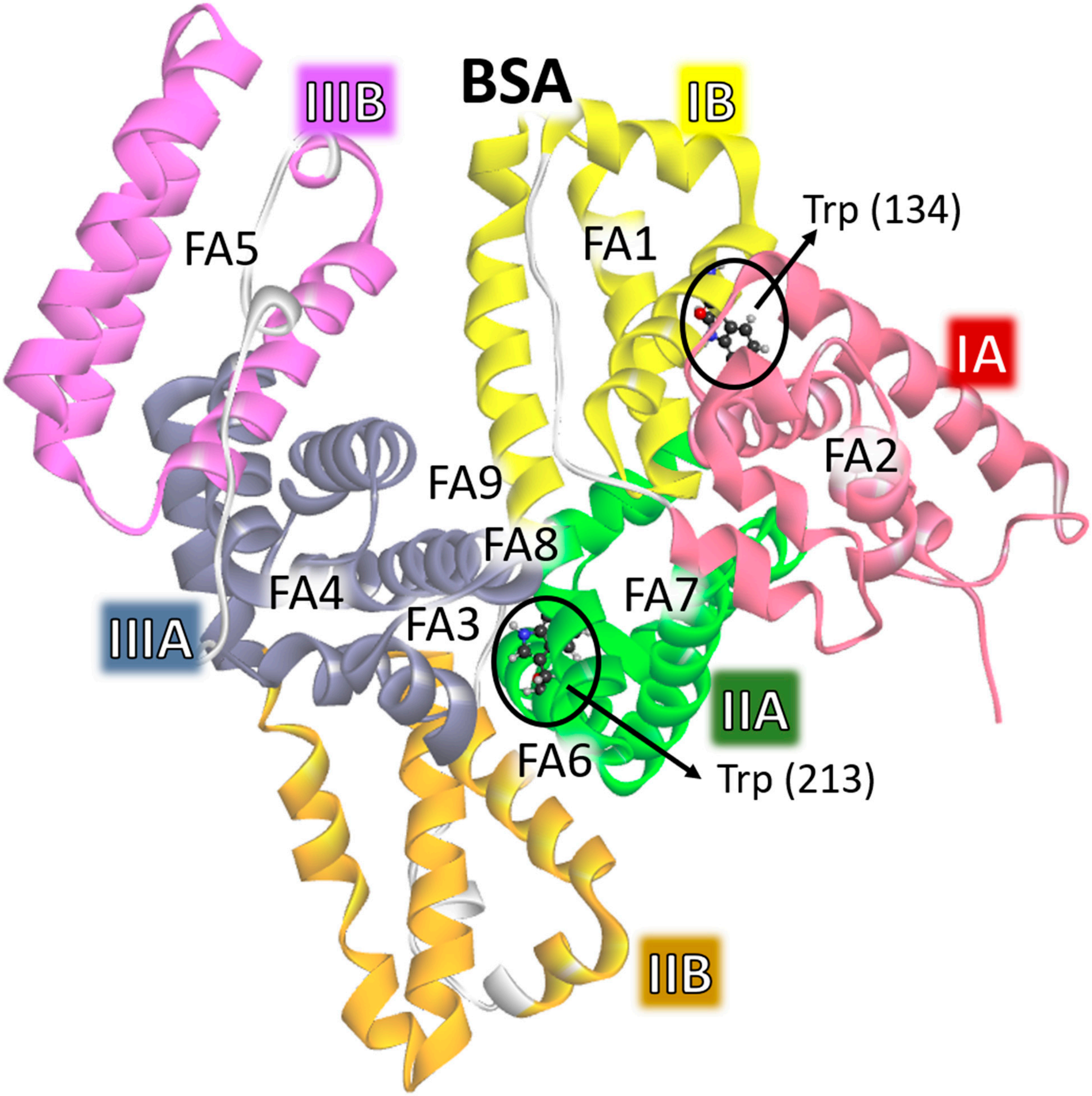

Figure S2. The crystal structure of BSA, with distinct coloring denoting its various sub-domains: IA (red), IB (yellow), IIA (green), IIB (orange), IIIA (dark gray), and IIIB (pink). Additionally, the binding sites—FA1 through FA9—are primarily intended for fatty acids but can also accommodate a range of other ligands, including drugs, minerals, and nutrients.
